# Acknowledgment to Reviewers of *Microorganisms* in 2020

**DOI:** 10.3390/microorganisms9020268

**Published:** 2021-01-28

**Authors:** Microorganisms Editorial Office

**Affiliations:** MDPI AG, St. Alban-Anlage 66, 4052 Basel, Switzerland

Peer review is the driving force of journal development, and reviewers are gatekeepers who ensure that *Microorganisms* maintains its standards for the high quality of its published papers. Thanks to the cooperation of our reviewers, in 2020, the median time to first decision was 15 days and the median time to publication was 34 days. The editors would like to express their sincere gratitude to the following reviewers for their precious time and dedication, regardless of whether the papers were eventually published:
Abate, GiuliaAhrens, ChristianAbdallah, AliAikawa, TakuyaAbdelfattah, AhmedAimanianda, VishukumarAbdelmohsen, Usama RamadanAime, MaryAbdel-Rahman, Mohamed AliAizpurua, OstaizkaAbera, BiresawAkad, FouadAbia, Akebe Luther KingAkins, RobertAbu-Niaaj, LubnaAlam, Md TauqeerAccardo, GraziaAlam, SaminaAccotto, Gian PaoloAlam, Seemi TasnimAcedo, Jeella Z.Alamo, TeodoroAceti, AntonioAlanio, AlexandreAcevedo, FernandoAlberdi, AnttonAchal, VarenyamAlberti, FabrizioAchinas, SpyridonAlbrecht, RandyAdadi, PariseAlbu, SilviuAdamakis, Ioannis-DimosthenisAlcendor, Donald J.Adamczyk-Popławska, MonikaAlduina, RosaAdams, VickiAleksandrzak-Piekarczyk, TamaraAdamus-Białek, WiolettaAlex, AnoopAdekunle, AdemolaAlexopoulos, AthanassiosAdewole, DeborahAlfieri, CarolineAdhikari, RajanAlfredsen, GryAdkar-Purushothama, Charith RajAli Zaidi, ShanAfrashtehfar, Kelvin IanAlias-Villegas, CynthiaAgeitos, Jose ManuelAlisaac, EliasAgnoli-Antkowiak, KirstyAlizadeh, HosseinAgoro, RafiouAlkasrawi, MalekAgrimonti, CaterinaAllaoui, AbdelmounaaïmAgyei, DominicAllsopp, Luke PAhern, PhilipAlmahli, HadiaAhmad, RafiAl-Marzooqi, WaleedAhmad, ShakilAltegoer, FlorianAhn, JuheeAltintas, Mehmet MAhn, Jung HoonAlunda, José MaríaAlvarez Fernandez, María AntoniaArenas, JesúsÁlvarez-López, VanessaArie, TsutomuAmaro, FranciscoAriño, AgustínAmat, SamatAriyawansa, Hiran AnjanaAmbrosino, ElenaArkadiusz, DziedzicAmemiya, KeiArnedo-Pena, AlbertoAmillis, SotirisArora, GunjanAmils, RicardoArosio, PaoloAmmendola, SerenaArrigoni, RobertoAmores Arrocha, AntonioArturo Vera Ponce De Leon, ArturoAmoroso, Maria GraziaArukha, Ananta PrasadAmund, DanielAscenzioni, FiorentinaAn, RanAschtgen, Marie-stephanieAnastasio, AnielloAshour, HossamAnco, Daniel J.Ashrafi, RoghaiehAnderson, Kirk E.Aslam, MueenAnderson, O. R.Aspatwar, AshokAndrade, JoséAspray, ThomasAndrade, María J.Assaf, Jean-ClaudeAndrás, IbolyaAstals, SergiAndreansky, SamitaAubert, RachaelAndree, Karl B.Audette, Gerald F.Andreev, KonstantinAuguste, Albert J.Andreolli, MarcoAugustyniak, DariaAndrianasolo, EricAulitto, MartinaÁngeles Lezcano, MaríaAung, Meiji SoeAngelici, Maria CristinaAurilia, VincenzoAngelidis, ApostolosAustin, BrianAngelino, DonatoAustriaco, NicanorAnghel, AndreiAvendaño-Ortiz, JoséAnne, JozefAvila-Román, JavierAnnenkova, NataliiaAvram, MarioaraANNIBALLI, FabrizioAvrani, SaritAnsaldi, MireilleAwasthi, MayankaAntolak, HubertAyayee, Paul AkwetteyAntonella, FarinaAyllon, AaronAntonelli, AlbertoAzab, WalidAntonets, KirillAzam, Aa HaerumanAntunes, Francisco José NunesAzarnia Tehran, DomenicoAntwerpen, MarkusAzcarate-Peril, AndreaAonofriesei, FlorinAzcona San Julian, María CristinaAprea, GiuseppeAzevedo, AndreiaAragona, MariaAzevedo-Pereira, José MiguelArana, InesAznar, Francisco JavierAranda, AgustínAznar, RosaArango Duque, GuillermoAzul, Anabela MarisaAraujo, RicardoBa, Hoa VanArbuckle, JesseBabenko, Vladislav V.Arbuthnot, PatrickBabu, GiridharArce-Fonseca, MinervaBaccile, NikiArdelean, Ioan I.Bach, BenoitBachmann, MartinBarnett, MeganBachmann, NathanBarnett, Stephen J.Bae, TaeokBarny, Marie-AnneBagetta, GiacintoBaron, Julianne L.Bagi, AndreaBarr, JohnBagiński, MaciejBarreca, DavideBagnoli, FabioBarreca, SalvatoreBahrim, GabrielaBarrett, AlanBai, S. C.Barszcz, MarcinBailey, Adam L.Bart, RebeccaBajaj, RuchikaBartas, MartinBaker, Jonathon L.Barth, ValdirBakre, Abhijeet A.Bartocci, PietroBalan, SreeKumarBartoli, FlaviaBalatskaya, Maria N.Bartolomé, BegoñaBalczun, CarstenBarton, Larry L.Baldassarre, Maria ElisabettaBartosik, KatarzynaBaldin, ClaraBartowsky, EvelineBaldomà, LauraBar-Zeev, EdoBalestrini, RaffaellaBasaglia, MarinaBalestrini, Raffaella MariaBasen, MirkoBallegeer, MarliesBashan, Luz E.Ballesté, ElisendaBasmaciyan, LouiseBallini, AndreaBastidas-Oyanedel, Juan-RodrigoBalogh, KrisztiánBasu, RupsaBaltz, RichardBatoni, GiovannaBan, BhupalBattistoni, AndreaBanach, ArturBatuman, OzgurBanadyga, LoganBawage, SwapnilBańbura, Marcin W.Bay, R. CurtisBandelt, Hans-JürgenBaymakova, MagdalenaBanki, OlafBazghaleh, NavidBankoti, KamakshiBazzicalupo, MarcoBanks, JonathanBeale, DavidBannantine, John PBeard, MatthewBannantine, John P.Beasley, ShannonBansal, Geetha P.Beasley, SheaBanta, ScottBeaumont, MartinBaptista, Mafalda S.Beaver, CarolBaptista, Rodrigo De PaulaBecker, JoyBara, JeffreyBecker, KarstenBarbieri, NicolleBecker, YvonneBarbosa, Joana Inês BastosBecker-Dreps, SylviaBarcena, CarmenBeedessee, GirishBar-Haim, ErezBehnke-Borowczyk, JolantaBarik, SailenBehrends, VolkerBarinova, SophiaBehzadi, PayamBaritugo, Kei AnneBej, AsimBarja, Juan L.Belanger, Faith C.Barkan, DanielBelbahri, LassaadBarlow, JohnBelbis, GuillaumeBeld, JorisBhattacharya, SomanonBell, RebeccaBiagini, GiuseppeBellanger, XavierBickford, Wesley A.Bellardi, Maria GraziaBierle, CraigBellini, MassimoBiernat, BeataBelloch, CarmelaBiernat, Monika MariaBeloborodova, Natalia V.Bilger, AndreaBelov, Andrey A.Bilitewski, UrsulaBeltran, GemmaBillamboz, MurielBełżecki, GrzegorzBillmyre, BlakeBenedec, DanielaBilska-Wilkosz, AnnaBenfaremo, DevisBingham, RichardBenini, StefanoBiolatti, MatteoBenítez-Páez, AlfonsoBiscotti, Maria AssuntaBenito, ÁngelBisha, BledarBenoit, MatthiasBitar, IbrahimBenucci, GianBivins, AaronBerchowitz, Luke E.Bjarnsholt, ThomasBerdan, EmmaBlacher, EranBerestetskiy, AlexanderBlagojevic Zagorac, GordanaBerg, JasmineBlaise, MickaelBerger, PetyaBlanda, ValeriaBergstrom, KirkBlank, RalfBerkelmann, DirkBlasco, LucíaBerlec, AlešBlasdell, KimBerlowska, JoannaBlasi, JuanBermudez, Luiz E.Blatzer, MichaelBernacchi, SébastienBleves, SophieBernotienė, RasaBloch, SylwiaBerriatua, EduardoBock, C. ThomasBertaccini, AssuntaBockmühl, DirkBertea, CinziaBodelier, PaulBertelloni, FabrizioBøgwald, JarlBerthelot, LaurelineBohacz, JustynaBerthold-Pluta, AnnaBojanowski, ChristineBertoglio, FedericoBolden, JayBertron, AlexandraBolshoy, AlexanderBertzbach, Luca DaniloBombelli, PaoloBerzal-Herranz, AlfredoBomberg, MalinBerzins, AivarsBon, Marie-claudeBessa, LucindaBonetta, SilviaBessaud, MaëlBonfield, Tracey L.Besson, BenoitBongiorno, DafneBesteiro, SébastienBorella, PaolaBetekhtin, AlexanderBorghese, RobertoBeumer, RijkeltBorghi, ElisaBevilacqua, AntonioBoroń-Kaczmarska, AnnaBeyer, MarcoBoros-Lajszner, EdytaBeyer, WolfgangBorras, EduardBezirtzoglou, EugeniaBorsetti, AlessandraBhattacharjee, SonaliBorsodi, Andrea K.Bortolami, AlessioBrown, Christopher LyonBörve, JorunnBrown, DennisBorza, TudorBruggemann, HolgerBoscari, AlexandreBrundage, Cord M.Boschiroli, María LauraBrune, WolframBosco, DomenicoBruns, TonyBosi, PaoloBryła, MarcinBosilevac, JosephBuan, NicoleBosis, EranBubici, GiovanniBostik, PavelBuchanan, RobertBotana, Ana MaríaBuckel, WolfgangBotelho, JoãoBudroni, MarilenaBotella, LeticiaBueno-Silva, BrunoBothner, BrianBui, Duc-CuongBotté, E. S.Buján Gómez, NoemíBotwright, NatashaBujňáková, DobroslavaBoucias, Drion G.Bulaev, AleksandrBouin, AlexisBulatov, EmilBourgeois, DenisBulatovic, MajaBovera, FulviaBulla Jr, LeeBoxman, IngeborgBültmann, HelgaBoyd, EricBuommino, ElisabettaBoyen, FilipBurger, Michael CBoyle, MariaBurgess, TreenaBoyom, Fabrice FekamBurkowska-But, AleksandraBoysen, ReinhardBuroni, SilviaBozue, JoelBurrai, Giovanni PietroBraks, MarietaBurrascano, JosephBrancaccio, MariaritaBurton, Jeremy P.Brassard, JulieBusby, Ryan R.Bratek, ZoltánBusco, GiovanniBraun, BurgaBuse, Helen Y.Brayton, Kelly A.Butaye, PatrickBréard, ÉmmanuelButkauskas, DaliusBrecht, MarcusButnariu, MonicaBrejnrod, AskerButtiglieri, GianluigiBrenciani, AndreaButtimer, ColinBresciani, ElenaButu, AlinaBrestic, MarianByadgi, OmkarBreton, LionelByrd, BrianBriandet, RomainByrne, AndrewBrierley, IanC. Benjamin, NamanBrígido, ClarisseCaballero, ArmandoBrilli, MatteoCabezas-Cruz, AlejandroBritt, Rodney D.Caeiro, Maria FilomenaBritz, Margaret L.Caetano, TaniaBrivio, MaurizioCaffrey, PatrickBroecker, FelixCagno, ValeriaBroom, Leon J.Cai, LeiBrown, BonnieCai, ShuoweiBrown, CharlesCal, Antonieta DeCalabrò, Paolo S.Cardoso, PauloCalasso, MariaCarducci, FedericaCalcutt, MichaelCarella, FrancescaCaldara, MarinaCarmeli, YehudaCalderaro, AdrianaCarradori, SimoneCalendar, RichardCarrasco, JaimeCalero Valdayo, Patricia MariaCarrera, MónicaCalero-Cacere, WilliamCarrilho, EuniceCalevo, JacopoCarrillo, Catherine D.Calistri, PaoloCarrillo, EugeniaCallaghan, DavidCarro, LauraCallegari, Maria LuisaCarro, LorenaCalvet, XavierCarrouel, FlorenceCamacho Chab, Juan CarlosCarter, KatharineCamacho, MaríaCartmill, Andrew D.Camargo, Samira Esteves AfonsoCaruso, GabriellaCambillau, ChristianCarvajal, Thaddeus M.Campanaro, StefanoCarvalho, FilipeCampbell, Ellsworth M.Casapullo, AgostinoCampos, Joana Araújo Alves DeCaser, MatteoCampos, Maria Jorge GeraldesCasero, María CristinaCanapa, AdrianaCasper, PeterCanário, JCassidy, JosephCaneiras, CátiaCastañeda-Chávez, María Del RefugioCanela, NúriaCastiglioni, BiancaCanganella, FrancescoCastillo, DanielCannon, CarolynCastro, HelenaCano, Israel GarciaCastro-Muñoz, RobertoCanoBuendía, José A.Cataldi, AngelCantalejo, María J.Catar, RusanCao, JianCatara, VittoriaCao, PengxingCattò, CristinaCao, ShengboCattoir, VincentCaparelli, RosannaCavalera, AlfonsaCaparros-Martin, JoséCayol, Jean-LucCapasso, RaffaeleCazacu, AnaCapdevila, Daiana ACeci, AndreaCapece, AngelaCeja-Navarro, Javier A.Capela, DelphineCelińska, EwelinaCapillo, GioeleCellini, AntonioCapozzi, VittorioCeranowicz, PiotrCaprai, ElisabettaCerbo, Alessandro DiCaputo, LeonardoCernava, TomislavCaputo, ValerioCervantes, JorgeCara, Francesca DiCervantes, MarcellaCarabetta, Valerie J.Cesbron, SophieCaradonia, FedericaChakaroun, Rima M.Cardarelli, MariateresaChalker-Scott, LindaCardazzo, BarbaraChamakura, Karthik R.Cardenia, VladimiroChan, JoshuaCardona, DianaChan, SunneyChandrashekar, AbishekChiarini, LuigiChang, EdwinChifiriuc, Mariana CarmenChang, Hao-XunChifiruc, Mariana CarmenChang, MatthewChilaka, CynthiaChang, Theresa Li-YunChiou, Chien-ShunChang, Wen-WeiChippaux, Jean-PhilippeChanway, ChristopherChistoserdova, LudmilaChao, YanjieChitlaru, TheodorChapeland-Leclerc, FlorenceChiu, Chia-YuChaplin, AliceChládek, GustavChapman, MatthewCho, Kyu HongChaptal, VincentChochlakis, DimosthenisCharusanti, PepChodosh, JamesChase-Topping, MargoChoi, Ki ChoonChatel, Jean-MarcChoi, MyeongjinChaudhary, Dhiraj KumarChoi, Youkyung H.Chauhan, NikhilChomel, BrunoChaves, Luis FernandoChong, Yong PilChaves, Susana RodriguesChopyk, JessicaChavez, Adela OlivaChorianopoulou, Styliani N.Chavez, ClemenciaChou, Lan-SzuChelsky, DanielChow, Franklin Wang-NgaiChen, GuijieChowdhury, NityanandaChen, Hui-WenChristensen, HenrikChen, JianwuChristodoulides, MyronChen, Kuan-FuChrzanowski, ŁukaszChen, LianminChu, Tin-ChunChen, LibenChua, BeeleeChen, Pin-jungChua, Eng GuanChen, Po-WenChubiz, LonChen, QiChul-sung, HuhChen, Shuen-EiChung, DonghoonChen, Wei-JuneChurchward, ColinChen, XianmingChurro, CatarinaChen, Yuan-ChuanCicatelli, AngelaChen, ZhaoCicciù, MarcoCheng, Chien-YuCiesielski, SlawomirCheng, HaoCieslak, AdamCheng, Jya-WeiCilia, GiovanniCheng, Kuan-ChenCipak, LubosCheng, LailiangCitterio, BarbaraCheng, Luisa W.Claessen, DennisCheng, Meng-ChunClark, MelodyCheng, XiaomingClark, Nicholas J.Cheng, ZishuoClark, Theodore G.Cheptsov, VladimirClark-Curtiss, Josephine E.Chern, Ming-KaiClaus, HaraldChernoff, YuryClay, KeithChiang, Chih-PoClement, CristinaChiang, Hsin-ICliffe, AnnaChiang, Yu-ChungCoates, Christopher J.Cobb, AdamCosoveanu, AndreeaCoburn, Phillip S.Cosseddu, Gian MarioCockburn, DarrellCostantini, AntonellaCodina, GeorgianaCostantini, LaraCoelho, Ana VarelaCostantino, ValeriaColdea, Teodora EmiliaCotârleț, MihaelaCole, JeffCote, ChristopherColebunders, RobertCoulibaly, Wahauwouele HermannColeine, ClaudiaCoutant, FrédéricColeman, DavidCowger, ChristinaCollier, AndrewCox, EricCollins, M.T.Craddock, Hillary A.Collins, MichaelCramer, BenediktColom Valiente, María FranciscaCrawford, LindseyColonna, BiancaCrenshaw, Joseph D.Coloretti, FabioCreusot, RemiComas-Garcia, MauricioCreuzenet, CaroleComitini, FrancescaCrevecoeur, SophieCompson, ZacchaeusCrispi, StefaniaCondemine, GuyCrnkovic, AnaConejeros, IvánCrocchiolo, RobertoCongestri, RobertaCroft, LarryConlon, MichaelCrognale, SimonaConn, David BruceCrombie, Andrew T.Conrads, GeorgCrook, BrianConsole, LaraCrossman, LisaConstantinescu-Aruxandei, DianaCruz Diaz, DavidConstantinos, PapagiannitsisCubero, JaimeContaldo, MariaCubillos, FranciscoContaldo, NicolettaCui, ZhengConti, PioCui, ZhouqiContreras, AngelaCulebras, EstherContreras-Cornejo, Hexon AngelCurtin, ChristopherConway, BrianCusco, AnnaCook, CharlesCusick, Kathleen D.Cooper, KerryCutrignelli, Monica IsabellaCopolovici, Dana MariaCwalina, BeataCoraça-Huber, Débora C.Cyplik, PawełCorcoran, JenniferCytryńska, MałgorzataCork, SusanCzajkowski, RobertCornelis, PierreD. Patil, MaheshCorona, MiguelD’Accolti, MariaCorral, PaulinaD’Andrea, Marco MariaCorreia Coelho, Ana CláudiaD’Ardes, DamianoCorrente, MarialauraD’Argenio, ValeriaCorrò, MichelaD’auria, EnzaCorte, LauraD’Ettorre, GabriellaCortegiani, AndreaDa Silva Dias, Igor AlexandreCortés, Maria PilarDa Silva, GabrielaCorzo-Leon, DoraDabert, MiroslawaCosby, DouglasD’Agostino, PaulDAGUE, EtienneDe Simone, ClaudioDahl, Jan-UlrikDe Simone, SalvatoreDakhlallah, DuaaDe Souza Moreira, FatimaDalebroux, Zachary D.De Souza, Danival JoséDalessandri, DomenicoDe Stefano, VitaDall’Aglio, CeciliaDe Vero, LucianaDalloul, RamiDe Vos, DanielDam, Chinh Thi MyDe Vrieze, MoutD’Amelio, RaffaeleDe Zotti, MartaDamiani, GiovanniDea-Ayuela, María AuxiliadoraDamiano-Forano, EvelyneDean, AndrewDanhof, Heather A.Deback, ClaireDaniela, JakšićDECOUSSER, Jean WinocDanielak, DorotaDee, Scott ADao Thi, Viet LoanDefez, R.Darvishi, FarshadDegani, OfirDas, SauravDegola, FrancescaDas, SubhaDeigner, Hans-PeterDas, UndurtiDel Caro, AlessandraDashti, YousefDel Corso, AntonellaDastogeer, KhondokerDel Fresno Sánchez, CarlosDatta, KausikDel Gallo, MddalenaDatta, RahulDel Gaudio, RosannaDaugelavicius, RimantasDelaplane, KeithDaugrois, Jean-HeinrichDelbarre Ladrat, ChristineDaveran-Mingot, Marie-LineDelbarre-Ladrat, ChristineDavid, JohnDelfino, Domenico V.David, Michael Z.Delfraro, AdrianaDavies, NigelDelgado Santander, RicardoDavis, David A.Dell’Oste, ValentinaDavolos, DomenicoDelorme-Axford, ElizabethDavydenko, KaterynaDelpeyroux, FrancisDe Andrea, MarcoDelwart, EricDe Cerain, Adela LopezDenesvre, CarolineDe Curtis, FilippoDeng, PengDe Francesco, GiovanniDeng, Zhi-LuoDe Francisco, PatriciaDenis, MartineDe Freitas, Ana Cristina Cardoso Freitas LopesDenizot, JéremyDe Jonge, CindyDenkova-Kostova, Rositsa Stefanovade Jonge, MarienDerome, NicolasDe Kievit, TeresaDeryabina, Yu I.De Koning-Ward, TaniaDesai, NiyatiDe La Calle, FernandoDesai, Umesh R.De La Rua Domenech, RicardoDescamps, DelphyneDe La Varga, HerminiaDescombes, PatrickDe Leo, FilomenaDeshpande, GauraviDe Loof, ArnoldDeviatkin, Andrei A.De Los Reyes, CarolinaDevirgiliis, ChiaraDe Lurdes Dapkevicius, MariaDevlin, PaulDe Miccolis Angelini, Rita MilviaDey, PriyankarDe Paiva, Cintia S.Deyrup, StephenDharmaraj, RajmohanDonham, KelleyDi Ciccio, PierluigiDonkersley, PhilipDi Lallo, GustavoDonnarumma, GiovannaDi Micco, PierpaoloDonoghue, HelenDi Profio, FedericaDorea, CaetanoDi Rosa, RobertaDosoky, NouraDi, PeterDott, Giovanni MarascoDiakou, AnastasiaDou, ZhichengDiamantopoulou, PanagiotaDoulberis, MichaelDiane Valeriano, ValerieDoulgeraki, AgapiDianez Martinez, FernandoDourtoglou, VassilisDiaz Fernadez, PabloDovgal, Igor VasilyevichDíaz Martínez, MargaritaDow, Coad ThomasDickenson, Nicholas E.Dow, MaxDickey, AaronDownie, J. AllanDicksved, JohanDrabczyk, AnnaDie, JoseDragoš, AnnaDieser, MarkusDraheim, Roger R.Dietl, Anna-MariaDreher, Mark L.Dietrich, IsabelleDreyfus, GeorgesDíez, José MaríaDrlica, KarlDimitrellou, DimitraDrogari-Apiranthitou, MariaDimkić, IvicaDrosinos, EleutheriosDimopoulou, MariaDrouin, PascalDinos, GeorgiosDrożdż, RyszardDiogo, PatríciaDrucker, MartinDionisio, GiuseppeDu Plessis, Heinrich WDiop, SetteDuarte, AidaDišlers, AndrisDuarte, MargaridaDix, RichardDuarte, RicardoDixon, Ronald ADuarte, Vinícius DDjerada, ZoubirDuarte, Vinícius SilvaDkhar, Hedwin KitdorlangDubnau, DavidDlauchy, DenesDuchêne, MichaelDo Paço, AnaDucker, WilliamDo, PaulaDuda-Chodak, AleksandraDoak, Thomas G.Dudek, KatarzynaDobay, OrsolyaDudley, Jaquelin P.Dobrovolny, Hana M.Duffy, Fergal JDobslaw, DanielDufossé, LaurentDocea, AncaDumonceaux, Tim J.Doceul, VirginieDuncan, MaraDoddapattar, PrakashDunfield, PeterDogra, PranayDunisławska, AleksandraDogra, ShaillayDunthorn, MicahDolfing, JanDurante-Rodríguez, GonzaloDomig, KonradDurazzo, MarilenaDominguez, RubenDurmic, ZoeyDonadu, Matthew GavinoĐurović, SašaDonaldson, JanetDutartre, HélèneDonelli, GianfrancoDutour, OlivierDutta, SanjuctaEndo, MakotoDuval, Raphaël E.Endoh, RikiyaDuvernell, David D.Engelen, AschwinDuzgunes, NejatEnglezos, VasileiosDwidar, MohammedEo, Soo HyungDyall-Smith, MikeEparvier, VéroniqueDylag, MariuszEri, RajaramanDymarska, MonikaEroglu, AbdulkerimDzhavakhiya, VitalyEscott, CarlosDzieciol, MonikaEskandarian, Haig AlexanderDziedzic, ArkadiuszEspinel-Ingroff, AnaDziejman, MichelleEspinosa Padrón, ManuelDziendzikowska, KatarzynaEsposito, SergioEbada, SherifEspuelas, SocorroEbbole, Daniel J.Esseili, Malak A.Ebeed, HebaEsteve-Romero, JosepEboigbodin, KevinEtxebeste, OierEbrahim, WeaamEuverink, Gert-JanEcke, ThorstenEvangelisti, EdouardEconomou, VangelisEvans, PaulEdit, FarkasEverest, PaulEdwards, Adrianne N.Evilia, CarynEdwards, MartinExbrayat, Jean-MarieEdwards, SimonEyice-Broadbent, OzgeEfremenkova, Olga VF. Abdallah, MohamedEgert, MarkusFabiszewska, AgataEggeling, LotharFactor-Litvak, PamEgyed, LászlóFaden, Howard S.Ekino, KeisukeFagerquist, CliftonEl Bairi, KhalidFagoonee, SharmilaEl Hage, CharlesFaherty, Christina S.El Khalloufi, FatimaFaia, Arlete MendesElaswad, AhmedFalconi, MaurizioElbeaino, TouficFaleiro, Maria LeonorElena, CriscuoloFalgenhauer, LindaEleni, TopalidouFalkinham III, Joseph O.El-Esawi, Mohamed A.Fallon, Ann MEliopoulos, Panagiotis A.Falzone, LucaElkins, KellyFan, Ming Z.Ella, BhagyarajFang, Shiuh-BinEllett, FelixFanizzi, Francesco PaoloEllis, Terri N.Fanning, SElmassry, Moamen M.Farage, MirandaEloit, MarcFarcasanu, Ileana C.Elolimy, Ahmed A.Fardeau, Marie-LaureEl-Seedi, HeshamFargnoli, MarioEmbers, MonicaFarone, Mary B.Emmett, BryanFarup, Per GEmpadinhas, NunoFarzaneh, VahidEnache, Mădălin-IancuFasciana, TeresaEnderle, RasmusFaso, CarmenFathi Abdallah, MohamedFonseca, FilomenaFaust, KarolineForsman, MatsFavennec, LoicForsyth, MarkFavia, GuidoFosso, BrunoFavresse, JulienFoster, JohnFay, DavidFoster, TimFederici, ErmannoFouillaud, MireilleFeher, TamasFouladkhah, AliyarFeldman, SteveFournier, Pierre-EdouardFelgueiras, HelenaFox, JulieFelicetti, TommasoFoxman, BetsyFeliciano, JoanaFradin, ChantalFenice, MassimilianoFraga, HugoFeniouk, BorisFraga, IreneFeodorova, Valentina AFragasso, MariagiovannaFerdes, MarianaFragkou, Paraskevi C.Fernández López, IvánFrampton, Rebekah A.Fernández, LucíaFranceschi, FrancescoFernandez, Teresa TórtolaFrancesco, SmedileFernández-Acero, Francisco JavierFranchi, ElisabettaFernández-Alarcón, ClaudiaFrancikowski, JacekFernández-Tomé, SamuelFranciosi, ElenaFerra, BartłomiejFrancisco, Alexandra E.Ferreira, Ana CristinaFranco, SandraFerreira, Manuel MarquesFrancolini, IolandaFerrer-Espada, RaquelFranken, PhillipFerrer-Gallego, RaulFranz-Sebastian, KrahFerrer-Gallego, RaúlFrasca, LoredanaFerrero, RichardFratini, FilippoFidalgo, CátiaFreimoser, FlorianFierro, José FernandoFreitas-Astúa, JulianaFilipello, VirginiaFrias Casas, MarioFilippidou, SevastiFritze, HannuFiliz, ErtugrulFrossard, Jean-PierreFimia, Gian MariaFry, LindsayFine, PaulFry, William EarlFinore, IlariaFu, XinyuFioretto, AntoniettaFuehrer, Hans-PeterFiorilli, MassimoFuentes, Màrius V.Fischer, Anthony J.Fujiwara, TaketomoFlament, Saskia C.Fukiya, SatoruFlanagan, DennisFukuda, KenjiFlannery, JohnFukuta, ShiroFleites, Laura A.Fuly, AndréFlemington, ErikFunari, RiccardoFlores-Félix, J.D.Funnell, SimonFloris, RosannaFurfaro, Lucy L.Flowers, PaulFurse, SamuelFogacci, FedericaFurtak, KarolinaFolch-Mallol, Jorge L.Furukawa, TakashiFolco, Hernan DiegoFuruse, YukiFusco, VincenzinaGargari, GiorgioGaardbo Kuhn, KatrinGarnett, JamesGabriel, IwonaGarrido-Fernández, AntonioGack, MichaelaGarrido-Maestu, AlejandroGaddam, Ravinder ReddyGarrido-Sanz, DanielGagat, PrzemyslawGarrill, AshleyGaglione, RosaGarsina, DanielleGagnevin, LionelGastmeier, PetraGajardo, GonzaloGatesy, John E.Gajdács, MárióGaul, DavidGajdosik, Martina SrajerGaylarde, ChristineGala, Rikhav P.Gay-Quéheillard, JeromeGalachyants, Agnia D.Gazzotti, StefanoGalan Ladero, Maria AngelesGeden, Christopher J.Galanis, AlexisGekenidis, Maria-TheresiaGalán-Sánchez, FátimaGell, David A.Galdiero, EmiliaGelli, AngieGaldiero, MassimilianoGelpi, MarcoGaliano, MonicaGeml, JózsefGalindo-Villegas, JorgeGenco, Caroline AttardoGallagher, JenniferGenereux, Diane P.Gallegos Monterrosa, RamsesGenovese, CarloGalli, ViolaGenovese, Kenneth J.Gallo, GaetanoGeorge, SantoshGalluzzi, L.Georgiou, SophiaGambarian, AlexandraGerbi, VincenzoGambley, Cherie F.Gerdol, MarcoGan, PamelaGerin, DonatoGAN, Yunn HwenGerlich, WolframGanaie, SafderGermini, DiegoGanatsios, VassiliosGershwin, LaurelGanesan, A.Gestal, Monica CartelleGanji, RakeshGhafar, AbdulGantner, MagdalenaGharsallah, HoudaGao, BeiGheorghiu, EugenGao, ChunxuGherman, Calin MirceaGaraizar Candina, JavierGhosh, ArnabGarbayo, InésGhosh, SumitGarcia Perez, SamuelGhoul, MelanieGarcía, MargaritaGiacomello, StefaniaGarcía, María JesúsGiammanco, AnnaGarcia-Descalzo, LauraGiannoni, FedericoGarcia-Jimenez, PilarGianotti, AndreaGarcía-Martínez, TeresaGiantsis, IoannisGarcia-Mazcorro, JoseGiaveno, AlejandraGarcía-Moyano, AntonioGill, HarsharnGarcía-R, Juan CarlosGilot-Fromont, EmmanuelleGarcıá-Sánchez, MercedesGino, SarahGarcía-Sánchez, SusanaGiovanardi, DavideGarcía-Suárez, María Del MarGiri, SibGardlik, RomanGiurg, MirosławGiuseppe, ArcangeliGoraj, WeronikaGkolfakis, ParaskevasGordon, David M.Głąbska, DominikaGoris, TobiasGladyshev, EugeneGormley, EamonnGlenn, WendyGorrell, RebeccaGlinkowski, WojciechGorres, Kelly L.Globisch, DanielGosciniak, GrazynaGlorieux, GrietGospodarek, JaninaGlukhov, EvgeniaGoss, Michael J.Glupov, VictorGoto, YasuyukiGnanasundram, Sivakumar VadivelGöttfert, MichaelGobin, IvanaGozalbo Rovira, Roberto VicenteGodfrey, Henry P.Grabau, Larry J.Godoy-Vitorino, FilipaGrabiec, AleksanderGoffinet, ChristineGragg, Sara E.Goh, Gerard Kian-MengGrainge, IanGoh, ShanGrandi, MonicaGohda, EiichiGrangeteau, CedricGolabi, MohammadGrant, David CGołaś, IwonaGrant, IreneGolba, SylwiaGrass, GregorGoldman, AlejandraGrassi, AusiliaGolec, PiotrGray, ChristophertGolemi-Kotra, DasantilaGrazia, Fortina MariaGolender, NatalyaGraziano, GiuseppeGollmann, GünterGreen, HyattGolomazou, EleniGreetham, DarrenGolomidova, Alla KGregorio, Simona DiGolubkina, Nadezhda A.Greigert, ValentinGolyshina, Olga V.Grieco, FrancescoGomes, AntonioGriffitts, Joel S.Gomes, Maria SaloméGrigorieva, Elvira V.Gomes, SóniaGrillon, AntoineGómez, Christian R.Grimaud, RégisGómez, FernandoGrimes, Darrell JayGómez-Brandón, MaríaGrinholc, MGomez-Gallego, CarlosGrishin, Alexander V.Gómez-Muñoz, María TeresaGrishkan, IsabellaGomez-Pastrana, DavidGros-Louis, FrançoisGomez-Silva, BenitoGrüngreiff, KurtGomoiu, IoanaGrunig, GabrieleGonçalves, A PedroGrywalska, EwelinaGonda, SándorGryzińska, Magdalena MariaGonzales-Barron, UrsulaGrządziel, JarosławGonzalez, Juan M.Grzesiak, JakubGonzález-Castro, Jose LuisGrześkowiak, ŁukaszGonzález-Cerón, LiliaGrzybek, MaciejGonzález-Fandos, María ElenaGrzymajlo, KrzysztofGonzalo, Diego GarciaGuan, JiewenGoodman, SteveGudiña, EduardoGoossens, KarenGuérardel, YannGuerin, FrançoisHam, Jun-SangGuerra, Alfredo J.Hamarsheh, OmarGuerra-Assuncao, AfonsoHammerl, Jens AndreGuerra-Sanchez, GuadalupeHammoudi Halat, DalalGugliandolo, ConcettaHan, YanGuidi, AlessandraHan, Yeon SooGuillamón, Jósé ManuelHancock, MeaghanGuillen Fretes, Rosa MariaHann, Hie-WonGuillén-Bejarano, RafaelHanson, BuckGuillotin, BrunoHanssens, LaurenceGuillou, SandrineHansson, IngridGullo, MariaHantke, KlausGunaje, JayaramaHao, GuixiaGunn, John S.Hara, HirofumiGuo, GraceHarada, KazukiGuo, Ming-LeiHarada, SoheiGuo, MingruoHarald, ClausGupta, CharuHarder, JensGupta, KuldeepHarding, MichaelGupta, SushimHardtke-Wolenski, MatthiasGupta, Vadakattu V.S.R.Hardwidge, PhilipGupta, VijaiHargreaves, IainGupta, VijayalaxmiHarrison, Lisa M.Gurrapu, SreeharshaHasan, ShakirGuskov, AlbertHaskey, NatashaGussago, CristinaHaslinger, KristinaGustafson, John E.Hassan, Sherif T. S.Gutiérrez, Ana RosaHassani, Mohamed-AmineGutiérrez-Gutiérrez, CarlosHauser, ElisabethGutiérrez-Martin, César B.Haverkamp, ThomasGutmann, MarcusHawman, DavidGuy, PaulHay, WilliamGuzik, MaciejHayashi, Jun-ichiroGuzzon, AntonellaHayes, Sidney J.Gyöngyvér, MaraHazlett, KarstenHaange, Sven-BastiaanHe, LixiaHaase, Elaine M.He, PeijianHack, EthanHeczko, PiotrHackshaw, KevinHeeb, StephanHać-Szymańczuk, ElżbietaHefferon, Kathleen L.Hadwiger, Jeffrey A.Hegazy, Mohamed-Elamir F.Hage-Ahmed, KarinHeinisch, Jürgen J.Haghshenas, Sami ShaffieeHeipieper, HermannHagi, TatsuroHeitman, JosephHahn, MartinHejnol, AndreasHahn, MatthiasHelena Siegel, MarciaHaile, JemaneshHelena, AlbanoHajra, AdrijaHelmy, Yosra A.Haley, BraddHenare, Sharon J.Halwachs, BettinaHenderson, Andrew J.Ham, Jong HyunHeney, ClaireHeng, XiaoHong, JinsuHengesbach, MartinHonjoh, Ken-ichiHenriques, Ana RitaHook, Lauren M.Herbig, AlexanderHoover, Timothy R.Heredero-Bermejo, IreneHooykaas, PaulHernáez, BrunoHoppert, MichaelHernández, María Del MarHorcajo Iglesias, PilarHernández-Rodríguez, CésarHorel, ÁgotaHerrero Fresno, AnaHorhat, Florin GeorgeHettinger, Jeffrey D.Horisawa, SakaeHeuner, KlausHornung, BastianHeyndrickx, MarcHorvath, AngelaHibberd, MatthewHorváth, Eszter MáriaHibbing, MichaelHorvathova, TereziaHibi, TakaoHorvatits, ThomasHietala, AriHoshino, TamotsuHiggins, N.Hotea, IonelaHiguchi, TomihikoHotos, George N.Hihara, YukakoHoughton, Karen MHildebrandt, Jan-PeterHoughton, Lesley A.Hill, FraserHouldcroft, CharlotteHillmann, FalkHoward, JonathanHilszczańska, DorotaHozawa, MikaHindra, HindraHrabar, JerkoHirai, HirofumiHranilovic, AnaHirai, NobumitsuHrazdilova, KristynaHiraishi, AkiraHrdy, JiriHirakawa, HidetadaHroncová, EmíliaHirakawa, YoshihisaHsieh, Ming KunHitchings, MatthewHu, ChenlinHnilicka, FrantisekHu, JiafenHo, MengchiaoHu, JieHodac, LadislavHu, JunhuiHoelzle, Ludwig E.Hu, QingHogan, DeborahHuang, DanweiHogsette, Jerome A.Huang, I-S. WadeHolatko, JiriHuang, Jason C.Holb, ImreHuang, JianshengHolbrook, MichaelHuang, JinlingHolden, James F.Huang, KaiHolden, NicolaHuang, Tse-HungHolert, JohannesHuang, WeihuaHolič, RomanHuang, XingfengHolland, MartinHuang, ZhuangrongHolmes, Anna D.Huang, Zuyi (Jacky)Holt, Scott M.Hubbard, MichelleHonda, YukiHubenova, YolinaHong, Bo-YoungHuck, OlivierHong, Chang PyoHudcovic, TomasHong, ChuanxueHudson, AndrewHong, Jiann-RueyHue, Erika S.Hughes, DiarmaidIseppi, RamonaHügle, BorisIshii, HideoHugouvieux-Cotte-Pattat, NicoleIshizuka, TakumiHulvey, Jonathan P.Islam, Md ZohorulHummel, MaryIslam, Md. TabibulHung, KennethIslam, Mohammad MazharulHung, Shao-WenIslam, Salim TimoHunt, Dana E.Ismaiel, AdnanHurtado, AnaIsokpehi, RaphaelHúska, DaliborIsola, GaetanoHussain, FatimaIzevbigie, Ernest B.Hussein, Islam T.M.Izquierdo-Bueno, InmaculadaHussein, MaythamIzuno, AyakoHusvéth, FerencJack, Thomas R.Hwang, You JinJackson, Sarah E.Hynes, Michael F.Jacob, PaulIacono, RobertaJagadeesan, BalamuruganIacumin, LucillaJagatić Korenika, Ana MarijaIanieri, AdrianaJagusztyn-Krynicka, ElżbietaIaniri, GiuseppeJaguva Vasudevan, Ananda AyyappanIannetta, MarcoJain, MitenIasur-Kruh, LilachJain, VaibhavIatsenko, IgorJaiswal, Amit KumarIbanez-Carrascoa, FreddyJakse, JernejIbekwe, Abasiofiok MarkJakubovics, NicholasIbrahim, FandiJalava, KatriIbrahim, SalamJames, Euan KevinIchinose, YukiJamil, TariqIerardi, EnzoJana, BimalIgnatov, AlexanderJanczarek, MonikaIkegami, TetsuroJanevska, SlavikaIliada, LappaJang, Kyoung-soonIlinskiy, YuriyJanga, Srikanth ReddyIlyasov, RustemJanganan, ThamaraiIm, JeongdaeJanik, KatrinImhoff, JohannesJanowicz, AnnaInglis, G. DouglasJanusz, GrzegorzIngram-Smith, CherylJarret, Robert L.Innocenti, NicolasJaskiewicz, MaciejInoue, ChihiroJaswal, RajneeshInoue, DaisukeJault, Jean-MichelInoue, JunJauregui, Rodrigo GutierrezInturri, RosannaJavǔrková, Veronika GvoždíkováIonescu, DannyJędryczka, MałgorzataIorizzo, MassimoJedziniak, PiotrIovino, FedericoJehmlich, NicoIpharraguerre, IgnacioJelocnik, MartinaIredell, JonathanJenvey, CaitlinIrrgang, AlexandraJeong, ChangyoonIsaguliants, MariaJeong, Do-WonIsakova-Sivak, IrinaJeong, Kwang C.Jeong, Rae-DongKaclikova, EvaJeong, Seok HoonKaczmarek, ŁukaszJeong, Seung-WooKaczorek, EwaJesenak, MilosKaehler, BenjaminJesudoss Chelladurai, JebaKageyama, DaisukeJeyanathan, JeyamalarKagot, VictorJeżewska-Frąckowiak, JoannaKahler, AmyJha, Ramesh K.Kahler, CharleneJhun, Byung WooKainulainen, Markus H.Jiménez Cantizano, AnaKaján, Győző L.Jimenez-Gomez, AlejandroKajihara, MasahiroJimenez-Rondan, FelixKalatzis, Panos G.Jimi, ShiroKaldhone, PravinJindo, KeijiKalenik, MarekJohannsen, EricKalicki, BolesławJohansen, AndersKalita, MichałJohansson, BjörnKalra, DivyaJohansson, Mats W.Kalyuzhnaya, MarinaJohnson, Casey N.Kamennaya, NinaJohnson, John AKamiloglu, SenemJohnson, NicholasKamitani, ShigekiJohnston, Peter R.Kampf, GünterJończyk-Matysiak, EwaKampmeier, StefanieJones, BrianKamysz, ElżbietaJones, JeremyKamzolova, SvetlanaJones, MarjorieKan, Chi-waiJordao, LuisaKan, JinjunJørgensen, Mikkel GirkeKancelista, AnnaJorrin, BeatrizKanda, TatsuoJoseph, LucyKandil, Omnia M.Joseph, SusanKandylis, PanagiotisJoshi, AmitKane, MelissaJoshi, Shivali S.Kanematsu, HideyukiJosue, DelgadoKanetis, LoukasJoyce, Michael A.Kang, ChanKyuJu, Sang-YhunKang, SanghoonJude, Brooke A.Kanginakudru, SriramanaJukic, MarkoKania, Stephen A.Jukić, MarkoKanike, NeelakantaJung Woo, JinKanipe, CarlyJung, ChuleuiKant, SashiJurado, M.M.Kao, Cheng-YenJurenas, DukasKaplan, ShaunaJuwadi, Praveen RaoKaradjian, GregoryJůzl, MiroslavKaramanou, SpyridoulaK. Bhunia, ArunKaranis, PanagiotisK. Hassan, MohammadKaraś, MagdalenaKaberdin, Vladimir R.Karcz, DariuszKabisch, JohannesKarig, DavidKačániová, MiroslavaKarim, ShahidKacienė, GiedreKarkowska-Kuleta, JustynaKarliński, LeszekKido, JunKarlsbakk, EgilKieffer, NicolasKarpinski, Tomasz M.Kieliszek, MarekKarpiński, Tomasz M.Kile, James C.Karus, AvoKim, Bong-SooKarvouniaris, MariosKim, Byoung SikKarwowska, MałgorzataKim, Chu-YoungKarwowski, BoleslawKim, Do-HyungKasahara, KazuyukiKim, Hak JoongKashif, MuhammadKim, Han-NaKashman, YoelKim, HeejungKasperkiewicz, KatarzynaKim, HeiKasuga, TakaoKim, HoonKaszowska, MartaKim, Hye OneKatanić, ZoranaKim, Hye YoungKathariou, SophieKim, I-TaeKato, YusukeKim, Jae-gyuKatoh, ToshihikoKim, Jae-SeokKatsiapi, MatinaKim, Ji HyungKatsifas, Efstathios A.Kim, JieunKatz, RichardKim, Kwang-sunKaur, RupinderKim, Yu-JinKaushik, PrashantKimura, HirokazuKavanagh, KevinKimura, KoujiKawada, Jun-ichiKinthada, LakshmanakumarKawamura, AkiraKirchberger, PaulKawasaki, KiyonoriKirchner, SebastianKayumov, AiratKireev, Igor I.Kazimírová, MariaKirienko, NatashaKe, Liang-YinKirker, Grant T.Keiffer, TimothyKirkland, PeterKeiper, Brett D.Kishore, Dr. UdayKeller, LanceKislitsina, OlgaKeller, PeterKiss, AnnaKellogg, ChristinaKiss, AttilaKemski, MeganKiss, JánosKenna, Dervla T. D.Kittl, SonjaKępa, MałgorzataKittler, SophieKerkhoven, EduardKlähn, StephanKerou, MelinaKlaile, EstherKerrouche, AbdelfatehKlassen, RolandKessel, DavidKlee, Silke R.Keyel, Peter A.Kleinsteuber, SabineKhan, Aman UllahKlepsatel, PeterKhan, MohamedKletzin, ArnulfKhan, Mohd MohsinKlewicka, ElzbietaKhan, NababKlimek-Ochab, MagdalenaKhatami, AmenehKlimina, Ksenia M.Khatri, VishalKlitz, WilliamKhurshid, ZohaibKlose, ThomasKidd, Stephen P.Klutsch, JenniferKniel, KalmiaKot, Anna MariaKnoop, KathrynKotta-Loizou, IolyKnoshaug, EricKoussis, KonstantinosKo, Kwan SooKouvelis, Vassili N.Kobae, YoshihiroKovač, TihomirKobayashi, NobumichiKovacs, RenatoKobayashi, TetsuroKovatcheva-Datchary, PetiaKobiler, OrenKowalczyk, PawełKobus, ZbigniewKowalska, BeataKocsis, BelaKowtharapu, Bhavani S.Kodama, YukiKozačinski, LidijaKoestler, Benjamin J.Kozak, BartoszKogut, MichaelKozak, ChristineKohler, AnnegretKrajewska-Wędzina, MonikaKoichi, HashimotoKrajka-Kuźniak, ViolettaKoide, Roger T.Kramer, Carolyn D.Koirala, RanjanKravchik, MichaelKokkinakis, EmmanouilKrawczyk, KrzysztofKokoszyński, DariuszKregiel, Dorota MariaKoksharova, Olga A.Kreis, Nina-NaomiKolasa, MichałKřen, VladimirKoldehoff, MichaelKretschmer, MatthiasKolobov, Alexandr AlexandrovichKrezel, AndrzejKolosov, PeterKrier, FrançoisKomaki, HisayukiKrismer, BernhardKomínek, PetrKrivoruchko, AnastasiiaKondo, SatoruKrnjaja, VesnaKondo, TomoKroeker, AndreaKong, HyungjoonKrömker, VolkerKönönen, EijaKrstanović, VinkoKonopelski, PiotrKruger, Jacob S.Konstantinidis, Theocharis G.Krumbholz, AndiKonto-Ghiorghi, YoanKrutova, MarcelaKontsevaya, IrinaKu, BonsuKopp, JulianKu, SeockmoKopprio, GermánKuban-Jankowska, AlicjaKoraimann, GüntherKubiak, KatarzynaKordowska-Wiater, MonikaKublanov, IlyaKorenblum, ElisaKubow, StanKorneev, DenisKucharski, JanKorshun, Vladimir A.Kudo, FumitakaKosakyan, AnushKudva, Indira T.Kosecka-Strojek, MajaKujawska, MałgorzataKosik-Bogacka, DanutaKumagai, HidekiKosinsky, Robyn LauraKumar, AjayKostecka, MalgorzataKumar, AmitKöster, WolfgangKumar, AnandKostoulias, XeniaKumar, AnujKostov, GeorgiKumar, AshokKostyushev, DmitryKumar, HirdeshKot, AnnaKumar, JitendraKumar, RameshLastauskienė, EglėKumar, SujeetLastochkina, OksanaKunicka-Styczyńska, AlinaLaterza, LucreziaKuo, Shu-ChenLattorff, H. Michael G.Kuppusamy, PalaniselvamLaudadio, EmilianoKuroda, MasashiLauersen, KyleKurosawa, NorioLauzi, StefaniaKuryk, LukaszLavieu, GregoryKusch, StefanLavoie, KathleenKüsel, KirstenLawrence, Jeffrey G.Kushima, Ryoji P.Lax, SimonKushkevych, IvanLay, ChristopheKuyukina, Maria S.Lazado, CarloKuzdraliński, AdamLazado, Carlo C.Kuźniar, AgnieszkaLazar, CatalinKwak, DongmiLázaro-Martínez, José LuisKwiatkowski, PawelLe Dréan, GwenolaKwiecień, IwonaLe Maréchal, CarolineKwiecińska-Piróg, JoannaLe Roy, CarolineKwok, Henry Hang FaiLeake, JonathanKwon, Oh-DeogLeal, LornaKyei, George B.Lean, Fabian Z. X.Kyriazakis, Ilias KyriazakisLea-Smith, DavidLa Spina, MichelangeloLeber, BettinaLaanto, ElinaLeberecht, MartinLabrou, NikolaosLebron, José AntonioLabroussaa, FabienLeclercq, AlexandreLacal, JesusLeclère, ValérieLacey, JakeLedford, Julie G.Lachance, Marc-AndréLee, A.-W.Lade, Harshad S.Lee, Chih-HsinLagatolla, CristinaLee, Chol GyuLahlali, RachidLee, ChoonghoLai, AlexanderLee, Dae-HeeLai, Michael M. C.Lee, Eun YeolŁakomy, PiotrLee, GihyunLalak-Kańczugowska, JustynaLee, Hae KyungLalmanach, Anne-ChristineLee, Je ChulLam, YanLee, Jin-HoLamas, AlexandreLee, Jong-HoonLammers, MichaelLee, Jung RoLandolfo, SantoLee, Keun HwaLanga, SusanaLee, Moo-SeungLanza, GiuseppeLee, NatuschkaLapaquette, PierreLee, Sae-ByukLaparra, Jose MoisésLee, Suk KyeongLaranjo, MartaLee, Tzu-TaiLarraga, VicenteLeewis, Mary-CathrineLarson, Marilynn A.Legrand, JackLarussa, TizianaLehman, John M.Larzábal, MarianoLehnherr, HansjörgLehtoranta, LiisaLin, Cheng-WenLeifels, MatsLin, SenjieLeimanis, MaraLin, ShaolingLeitão, AnaLin, XiaorongLeitão, Jorge H.Lin, Yi-TsungLeitgeb, MajaLin, Yu-PinLelešius, RaimundasLinaldeddu, Benedetto T.Leliaert, FrederikLinares-Pastén, Javier A.Lenart-Boroń, AnnaLinciano, PasqualeLenhard, Justin R.Lindahl, LasseLeone, PhilippeLindemann, Stephen R.Leon-González, Antonio J.Lindoso, José Angelo LaulettaLeoni, ClaudiaLing, Maurice H. T.Lepczyński, AdamLinial, MichalLeplat, JohannLinke, DirkLeppänen, Hanna K.Lipińska, AndreaLeppla, StephenLisandru, CapaiLeroy, FredericLiss, Steven N.Lesueur, DidierLiu, Chao-LinLetek, MichalLiu, ChaoxingLetelier, RicardoLiu, Chung-JungLetoha, TamásLiu, ChunyuLettini, Antonia AnnaLiu, HongxiangLeveau, JohanLiu, Hsin-FuLevy, JerroldLiu, JianmingLeyuan, LiLiu, Jun-HongLhomme, SébastienLiu, RongmingLi, BowenLiu, SaifeiLi, DongmeiLiu, TongLi, JiaweiLiu, WeiLi, KefengLiu, YanhongLi, Mei-LingLiu, Yung-ChuanLi, MingjiLiu, ZhongweiLi, RobertLjubin-Sternak, SuncanicaLi, TianhuLlarena, Ann-KatrinLi, WeiqiangLlorens, EugenioLi, WenyiLlorente, FranciscoLi, Xian-ZhiLo Giudice, AngelinaLi, Yu-YouLo, Hsiu-JungLiakopoulos, VassiliosLo, SzechengLiaw, Shwu-JenLobato-Marquez, DamianLibante, VirginieLobocka, MalgorzataLibich, DavidŁobocka, MalgorzataLiburdi, KatiaŁobocka, MałgorzataLicciardello, GraziaLoginov, DmitryLiebman, MichaelLoibner, Andreas P.Liechti, George W.Loimaranta, VuokkoLiegner, KennethLojkowska, EwaLiévin-Le Moal, VanessaLoman, Brett R.Lilleri, DanieleLombardi, TommasoLima, Bruno P.Lonardo, AmedeoLoncaric, IgorMackenstedt, UteLong, QuanMackenzie, JasonLongnecker, KristaMacone, AlbertoLoniewski, IgorMacori, GuerrinoŁoniewski, IgorMaddalone, MarcelloLoo, Xiu Ling EvelynMadeira De Carvalho, Luis ManuelLopes, Ana Patrícia AntunesMadison-Antenucci, SusanLopes, Lucas DantasMadrid, CristinaLopez Seijas, JacoboMaeda, YuichiLopez, Blanca R.Maehara, ShojiLópez, Javier RodríguezMaekawa, ShunLópez-Gómez, José PabloMaeseneire, Sofie DeLops, FrancescoMagariños Ferro, BeatrizLorenz, AlexanderMagierowski, MarcinLorenzo-Gómez, Maria-FernandaMagiorkinis, EmmanouilLorenzo-Morales, JacobMagnano Di San Lio, GaetanoLoreti, StefaniaMagri, GiulianaLoria, Guido RuggeroMahapatra, Ajit K.Losurdo, GiuseppeMaicas, SergiLouzao Ojeda, M. CarmenMaita, MasashiLowe, James F.Maixent, Jean MichelLower, BrianMakarewicz, WojciechLozano, Jose ManuelMakena, Monish RamLu, Ming-WeiMaki, JamesLu, YouMakris, AntoniosLucchino, BrunoMakvandi, PooyanLucey, BrigidMakwana, NandiniLücke, Friedrich-KarlMalacrinò, AntoninoLücker, SebastianMalagon, FranciscoLukashevich, IgorMaldonado, JuanLukaszewicz, MarcinMalec, PrzemysławLukomska-Szymanska, MonikaMaleki Vareki, SamanLumini, EricaMalerba, GiovanniLupușoru, RalucaMalfeito-Ferreira, ManuelLuster, Douglas G.Malgieri, GaetanoLutter, ErikaMallo, NataliaLuvisi, AndreaMaloberti, AlessandroLux, RenateMalwal, Satish R.Lux, ThomasMamo, GezahegneLuzzatto, TalMan, AdrianLynch, TarahManageiro, VeraMa, KesenMancabelli, LeonardoMa, Zheng FeeiMancianti, FrancescaMaack, ChristianMancini, InesMaaß, SandraMandal, ChanchalMacArthur, IainMandyam, KeerthiMacedo, Maria FilomenaManfrin, AmedeoMacGowan, Alasdair P.Mangiaterra, GianmarcoMachado, DianaMangot, Jean-FrançoisMachado, IdalinaMani, AlirezaMaciver, SutherlandManjunath, ManuboluManolescu, Loredana Sabina CorneliaMartinez-Gil, LuisManservigi, RobertoMartinez-Gomez, Norma CeciliaManti, SaraMartini, DanielaMao, MengMartinotti, SimonaMarc, DanielMartín-Peláez, SandraMarć, Małgorzata AnnaMartín-Rodríguez, Alberto J.Marçais, BenoitMartins Da Costa, PauloMarček, TihanaMartins, NataliaMarcer, FedericaMartin-Vicente, AdelaMarcheggiani, StefaniaMartorana, Alessandra M.Marchi, SerenaMarutescu, LuminitaMarciniak-Łukasiak, KatarzynaMaruyama, JunkiMarcone, Giorgia LetiziaMarx, ChrisMarczak, MałgorzataMarzec, MichałMarek, AgnieszkaMarzi, AndreaMarek, KieliszekMasalova, Olga V.Mareković, IvanaMashruwala, AmeyaMareș, MihaiMassad, L. StewartMaresca, MarcMassaglia, GiuliaMargalef, MariaMassart, SebastienMargassery, Lekha MenonMast, YvonneMargolles, AbelardoMastanjević, KrešimirMaria Balestrini, RaffaellaMastore, MaristellaMaria Teresa, FrangipaneMastroleo, FeliceMaria, PapaleMasullo, MariorosarioMárialigeti, KárolyMatei, FlorentinaMarichann, Manukumar HonnayakanahalliMateo, EstibalizMarin Lopez, AlejandroMathai, PrinceMarín, ClaraMathias, RommelMarín, María LuisaMatilla, MiguelMarino, TizianaMatsumoto, YasuhikoMario, NosottiMatsuoka, HirokiMarkopoulos, Georgios S.Matsushita, ShuzoMarkovska, RumyanaMatsuura, NorihisaMarkt, RudolfMattila, SariMarotta, FrancescaMattoscio, DomenicoMarotta, FrancescoMatucci Cerinic, MarcoMarová, IvanaMatusali, GiuliaMarreiros, BrunoMatys, JacekMarruchella, GiuseppeMaugeri, Andrea GiuseppeMars, Ruben A.Maunula, LeenaMartău, Adrian GheorgheMaupin-Furlow, Julie A.Martchenko, MikhailMaurer-Stroh, SebastianMartellos, StefanoMaurin, MaxMartenot, ClaireMaus, IrenaMartín Castillo, MargaritaMay, Kerrie L.Martín, AlbertoMayfield, Anderson B.Martin, StephenMayne, PeterMartinez, BeatrizMayo, ChristieMartínez-Cañamero, MagdalenaMayo-Prieto, SaraMartínez-Espinosa, Rosa MaríaMayumi, DaisukeMazurek-Popczyk, JustynaMeziti, AlexandraMcAllister, TimMezule, LindaMcCarthy, AlexMicael, JoanaMcCauley, MarkMichiels, ChrisMcClelland, ErinMichotey, ValerieMcCoy, Karen D.Middelveen, MarianneMcDonald, DanielMidwinter, AnneMcFarlane, Donald A.Miele, Adriana E.McFeeters, RobertMierzejewska, JolantaMcGill, JodiMigheli, QuiricoMcGiven, JohnMiglietta, MariaMcgregor, NeilMiglio, AriannaMcInroy, John A.Mihai, MaraMcIntosh, MatthewMihál, IvanMcLaughlin, JohnMihalca, Andrei DanielMcLucas, BruceMihasan, MariusMcNamara, PaulMi-ichi, FumikaMcNaughton, AnnaMikaelyan, Arsen S.McSharry, Brian PatrickMikolajczyk, AnitaMcSkimming, Daniel IanMilanova, AneliyaMcVicker, GarethMilhano Santos, FátimaMcVoy, MichaelMiller, Aaron W.McWhorter, AndreaMiller, Elizabeth M.Mead, Jan R.Mimuro, HitomiMedo, JurajMinami, MasaakiMehariya, SanjeetMiniero, Daniela ValeriaMehta, GunjanMira, Nuno P.Meibom, AndersMiranda-Sanchez, FabiolaMeiklejohn, Kelly A.Mirete, SalvadorMejias, AsunciónMirleau, PascalMelik, WessamMirończuk, Aleksandra M.Meliopoulos, Victoria A.Mir-Sanchis, IgnacioMellata, MelhaMishra, MeerambikaMelo, LauroMishra, NigamMelville, StephenMitchell, JenniferMena, AnaMitchell, JudyMendes, Marta V.Mitrea, Ioan LiviuMendis, Hajeewaka C.Miura, NatsukoMenendez, EstherMiyaji, AkimitsuMeneses, CarlosMiyajima, ToshihiroMenon, RaniMiyata, NaoyukiMerah, OthmaneMiyoshi, Shin-ichiMERCIER, CorinneMiyoshi, Shin-IchiMertas, AnnaMiyoshi-Akiyama, TohruMešić, ArminMłynski, DariuszMesnage, RobinMoar, WilliamMesquita, JoãoMoawad, Amira A.Messaritakis, IppokratisMocarski, EdwardMestres, ConxitaModica, MariaMeštrović, TomislavMoiseenko, Konstantin V.Metras, RaphaelleMojsoska, BiljanaMojumdar, KamalikaMorse, DanielMOK, Chee KengMorse, DavidMoldovan, Radu-CristianMorsy, MustafaMoles, LauraMos, BenjaminMolina Salinas, Gloria MaríaMoscati, LiviaMolina, RicardoMoscone, DanilaMolinero-Ruiz, LeireMoshiri, ArfaMolla, GianlucaMoss, Anthony G.Molmeret, MaëlleMossialos, DimitrisMołoń, MateuszMotherway, Mary O’connellMoloney, Gerard M.Motyka –Pomagruk, AgataMomotani, EiichiMouga, TeresaMoncalián, GabrielMouncey, Nigel J.Mondal, Utpal KumarMožina, Sonja SmoleMoniuszko, HannaMucciarelli, MarcoMonokrousos, NikolaosMucha, JoannaMonroy, FernandoMueller, Rebecca C.Montagnoli, AntonioMukherjee, MaitreyeeMontanari, ChiaraMukhopadhyay, TuliMonteagudo, AndreaMukhortova, LiudmilaMontero-Calasanz, CarmenMuller, MarcMoore, James D.Mulloy, BarbaraMoossavi, ShirinMumcuoglu, Kosta Y.Mora, DiegoMunch, Jean CharlesMorais, Livia HeckeMunekata, PauloMorales, DianaMuniesa, MaiteMorales-González, José AntonioMunk, CarstenMoreau, MagaliMuñoz-Muñoz, José LuisMoreira, Ana CarolinaMuntyan, Maria S.Moreira, Paula MariaMunyaka, Peris M.Morel, Chantal M.Munzi, SilvanaMoreno, Antonio D.Murakami, TakumiMoreno, EstherMurata, ShiroMoreno-Rojas, José ManuelMuratova, AnnaMorfin, NuriaMuravyov, MaximMorgan, EthanMurillo, JesusMorgan, NoelMurovska, Modra F.Morgunov, IgorMurphy, Caroline S.Mori, HiroshiMurphy, CormacMori, HirotadaMurphy, HelenMori, TetsushiMurray, PaulMorikawa, KazuyaMurrell, ColinMorimura, ShigeruMurtaza, NidaMorio, FlorentMusalgaonkar, SharmishthaMorita, YujiMusch, MarkMoriuchi, HiroyukiMustapha, AzlinMorley, KristaMuszewska, AnnaMorozov, IgorMuszyński, SiemowitMorozova, OlgaMuth, TheodoreMorozova, Vera V.Mutiga, Samuel K.Morroni, GianlucaMymryk, JoeMysliwiec, TamiNeubeck, AnnaMyung, HeejoonNeuhaus, KlausNa, DokyunNeumann, AnkeNaber, KurtNeumann, ArianeNacke, HeikoNeundorf, InesNadai, ChiaraNeustupa, JiříNadal, MarcNewton, Irene L. G.Nadǎş, George CosminNgamson, BongkotNagafuchi, SeihoNguyen, TrieuNaganuma, TakeshiNguyen, Van KhanhNagaoka, IsaoNicolas, PierreNagarajan, Uma M.Nicoletti, RosarioNagata, ToshiNicosia, AldoNagel, RaimundNiedbała, GniewkoNagpal, RavinderNiederwerder, Megan C.Naguib, Mahmoud MohamedNiedziela, TomaszNagy, NadineNiedźwiedzka-Rystwej, PaulinaNaka, HiroakiNielsen, BirtheNakahara, TomomiNielsen, Eva M.Nakajima, MasamiNieto, Marcelo J.Nakazawa, MasamiNiewiadomska, AlicjaNamsaraev, ZorigtoNigam, DeeptiNannipieri, PaoloNikoh, NaruoNanno, MasanobuNikolaivits, EfstratiosNardi, TizianaNikoloudakis, NikolaosNarouei-Khandan, Hossein A.Nishikawa, JunNarra, Hema PrasadNishiuchi, YukikoNasheri, NedaNissapatorn, VeeranootNastuta, Andrei VasileNissen, LorenzoNath, ArijitNissilä, EijaNathanailides, CosmasNiu, DongyanNatvig, Donald O.Nizza, AntoninoNavone, LauraNoecker, CeciliaNazik, HasanNoel, Samantha J.Nazina, TamaraNogales, AitorNeag, Adriana MariaNogueira, AntónioNeag, Maria AdrianaNoiriel, AlexandreNedoluzhko, ArtemNorén, TorbjörnNeelakantan, PrasannaNoszczyńska, MagdalenaNeffe-Skocińska, KatarzynaNovák, DavidNegut, IrinaNovikov, AndreiNehela, YasserNowakiewicz, AnetaNejman-Faleńczyk, BożenaNowakowska, JustynaNelsen, Matthew P.Ntaikou, IoannaNemec, AlexandrNtemiri, AlexandraNémeth, ÁronNtougias, SpyridonNero, LuisNunes, Márcio R.Nerva, LucaNurjadi, DennisNestares Pleguezuelo, Maria TeresaNurtop, ElifNetrusov, AlexanderNyman, DagNeubauer, KatarzynaO’neill, Catherine A.Oancea, FlorinOprea, OvidiuObrenovich, MarkOren, AharonObrenovich, Mark E. M.Orkusz, AgnieszkaOcci, JamesOrlić, SandiOchoa-Leyva, AdrianOrruño, MaiteO’Connell, CatherineOrsini, MassimilianoOda, MasatakaOrth, KimOdendall, CharlotteOshkin, Igor Y.Odermatt, Pascal D.Osorio, CarlosOdeyemi, Olumide A.Ostash, BohdanOdoch, TerenceOszako, TomaszOechslin, FrankOtalora, MonicaOelschlaeger, PeterOts, KatriOesterle, PaulOtulak-Kozieł, KatarzynaOgorek, RafalOughton, MatthewOgórek, RafałOukarroum, AbdallahO’Gorman, DavidOuwehand, ArthurOgunade, IbukunOuwendijk, Werner J. D.Ogunjirin, AdebowaleOyelade, TopeOh, Seung-YoonOzga, AndrewOhbayashi, TsubasaOzimek, EwaOhta, HiroshiOzkan, JeromeOhyama, TakujiÖztürk, BasakOishi, KenjiPace, FabioOkada, EisakuPachathundikandi, Suneesh KumarOkamoto, ShigefumiPacifico, DavideOkamura, YoshikoPaduszynska, MalgorzataOkech, Bernard A.Paetzold, BernhardO’Keefe, RaymondPagano, MarcelaOkino, TatsufumiPaholcsek, MelindaOkoń, SylwiaPais, PedroOkorski, AdamPakostova, EvaOlaru, Octavian TudorelPakpour, SepidehOlech, MonikaPal, ChandanOlejníková, PetraPal, SangitaOleskin, Alexander V.Palacios, José-ManuelOlga, PapadopoulouPalazzo, AntonioOliveira, ManuelaPaley, Elena L.Oliveira, RaquelPallecchi, LuciaOliveira, Rui S.Pallen, MarkOlivier, ChrystelPalma, Paulo JorgeOlivotto, IkePalmer, AndrewOmar, Ahmad A.Palsson, BernhardOmar, Syed HarisPanadero, RosarioOndreičková, KatarínaPanagou, E.Z.Ongerth, JerryPance, AlenaOniga, Smaranda DafinaPanda, SantoshOnisto, MaurizioPandit, SantoshOnodera, TakashiPane, CatelloOosterkamp, Margreet J.Pangeni, RudraOpoka, WlodzimierzPannella, GianfrancoPanstruga, RalphPatrick Lajoie, Patrick LajoiePanyushkina, AnnaPatrone, VaniaPaola, DolciPatsios, SotirisPaolino, GiovanniPaul, KimberlyPapa, FedericoPauleta, SofiaPapadeli, MarinaPaulino-Lima, Ivan GláucioPapadimitriou, TheodotiPavelko, Kevin D.Papadopoulos, GeorgiosPavic, ValentinaPapadopoulou, ChrissanthyPawar, KamleshPapagiannitsis, Costas C.Pawar, Shrikant DPapale, MariaPawlak, AleksandraPapanikolaou, EleniPawlik, AnnaPapanikolaou, SeraphimPayami, HaydehPapathanakos, GeorgiosPayne, MatthewPapp, JohnPayot-Lacroix, SophiePapp-Wallace, Krisztina M.Pazdzior, KatarzynaParamythiotis, SpirosPearlman, Ronald EdwardParente, EugenioPearson, AngelaPariani, ElenaPecar, DarjaParikesit, Arli AdityaPeck, RonaldPark, Hee-SooPecka-Kiełb, EwaPark, Hyun-WooPecyna, PaulinaPark, Kui-youngPeery, RhiannonPark, Myeong SooPei, YonggangPark, Sang WookPeiretti, Pier GiorgioPark, Se ChangPeiris, MadushaPark, Seon-JooPekala, AgnieszkaPark, Soo JePekkala, SatuPark, Youn ShikPellegrini, MarikaPark, Young-kyoungPeltola, VillePark, Young-SeoPeluso, IlariaParkar, Shanthi G.Pembroke, J.TonyParlati, LuciaPennerman, KaylaParsons, CameronPepe, TizianaParthasarathy, AnutthamanPepi, MilvaParveen, NikhatPerchec Merien, Anne-MariePascale, AlbertoPereira, Ana LuisaPashikanti, SrinathPereira, Cidália AlmeidaPashov, AnastasPereira, FilipePasqua, MartinaPerelomov, Leonid V.Passera, AlessandroPeretz, AviPassoth, VolkmarPérez García, Luis AntonioPastor, Sandra GarcésPerez-Casal, JosePastorino, PaoloPérez-Cobas, Ana ElenaPátek, MiroslavPerez-Pascual, DavidPatel, BhargavPérez-Tanoira, RamónPatel, HardikkumarPericchi, LuisPatel, Jai SinghPerin, GiorgioPaterson, Gavin K.Pero, RaffaelaPathak, AshishPeron, GregorioPatrauchan, Marianna A.Perpetuini, GiorgiaPerret, XavierPlacido, AntonioPerrot, TaraPlacido, JerssonPerrucci, StefaniaPlassard, ClaudePersad, Anil K.Plassard, Claude S.Persechino, SeverinoPlatt, ThomasPersiani, Anna MariaPlaza-Díaz, JulioPerugino, GiuseppePleckaityte, MildaPessi, TanjaPlessas, StavrosPester, MichaelPletzer, DanielPetar, PujićPlotka, MagdalenaPeterbauer, ClemensPlugge, CarolinePeterson, Eric CharlesPobiega, KatarzynaPetit, ThomasPodar, MirceaPetri, Renée MaxinePodgornik, AlešPetříčková, KateřinaPodleśny, JanuszPetrov, KaloyanPodnecky, Nicole L.Petrova, MayyaPodust, Larissa M.Petrova, PenkaPodzimek, StepanPetrovan, VladPöggeler, StefaniePetruzzi, LeonardoPokrovsky, Oleg S.Pfliegler, Walter P.Polak-Berecka, MagdalenaPflügl, StefanPoli, AnnaPhalak, PoonamPoli, AnnaritaPhee, LynettePolimeno, LorenzoPhilipp, BodoPolkowska, ZanetaPiatek, JacekPollastro, StefaniaPicchianti-Diamanti, AndreaPollmann, KatrinPichon, MaximePolo, JavierPieckova, ElenaPoltronieri, PalmiroPiedade, Ana PaulaPoma, AdolfoPielech-Przybylska, KatarzynaPomares, ChristellePierce, ElizabethPomari, ElenaPierce, EllenPomilio, FrancescoPietri, JosePons-Salort, MargaritaPietrini, IlariaPontejo, Sergio MPietrzyk-Brzezinska, Agnieszka J.Poole, ToniPilarska, Agnieszka A.Pop, Oana LeliaPiñeiro, LuisPop, Tudor LucianPinheiro, JoaquinaPopa, Mircea IoanPinto, Diana CláudiaPopham, David L.Pinto, LorisPorcel, RosaPinto, Pablo MartinPorcelli, FernandoPinto, SandraPorollo, AlekseyPires, AldaPorter, Abigail W.Pirhonen, MinnaPotaniec, BartłomiejPisarenko, SergeyPotekhin, AlexeyPiscitelli, PriscoPotrykus, MartaPiškur, BarbaraPoupard, PascalPita, LuciaPous Rodríguez, NarcísPitarch, AidaPowell, MarkPivetta, EmanuelePoyntner, CarolinePozzi, CeciliaQuinto, Emiliano JoséPrada, Christopher FernandezQuiquampoix, HervéPrakash, DivyaRaabe, Vanessa N.Pratscher, JenniferRABAH, HouemPrearo, MarinoRabito, Felicia A.Préat, AlainRachel, MartaPrendeville, Holly R.Radcliff, Fiona JanePrentis, PeterRafieenia, RaziehPrescott, GrahamRago, DanielaPresentato, AlessandroRagupathi, Naveen Kumar DevangaPrice, PatriciaRagusa, AndreaPrice-Carter, MarianRahbar, AfsarPrieto, DanielRahimi, ParvanehPrigione, ValeriaRahlff, JaninaPriyadarshini, MedhaRahman, AzizurProbst, MaraikeRahman, M. AzizurProchazkova, PetraRahman, Masmudur M.Proctor, Lita M.Rahman, MotiurProctor, RichardRai, Krishan MohanProença, Diogo NevesRai, NavneetProft, ThomasRaimondi, LaviniaProkhorov, NikolaiRaina, SatishPronin, Alexander V.Raisanen, RiikkaProvenzano, DanieleRajaiya, JayaProverbio, DanielaRajasekharan, Satish KumarPruess, Birgit M.Rakicka-Pustułka, MagdalenaPtaszynska, AnetaRakov, AlexeyPu, YuntingRakus, KrzysztofPucciarelli, SandraRamachandran, ReshmaPucker, BoasRamachandran, VinoyPukalchik, MariiaRamakrishnan, ChandraPulavarti, Surya VSRKRamakrishnan, SrinivasanPulcini, DomitillaRamamoorthy, AyyalusamyPulido Fernández, ManuelRamchandani, DivyaPurahong, WitoonRamesh, Chatragadda H.Puri, AkshitRamírez Soto, Max CarlosPurswani, JessicaRamírez, Ana SofíaPutignani, LorenzaRamirez-Castrillon, MauricioPytlak, AnnaRamirez-Malule, HowardPyzik, AdamRamos, SóniaQi, ChaoRamos-Garcia, PabloQin, HuaRamos-Vivas, JoséQu, BingqianRampacci, ElisaQuadeer, Ahmed A.Rampersad, SephraQuaglio, FrancescoRandazzo, WalterQuéméneur, MarianneRanjan, KishuQuéré, PascaleRantakokko-Jalava, KaisuQuero, Amparo JimenezRao, ChristopherQuessy, SylvainRapone, BiagioQuigley, EamonnRappo, UraniaQuilez, JoaquinRaquel, BecerrilRasmussen, Torben SølbeckRibaldone, Davide GiuseppeRassow, JoachimRibeiro-Barros, Ana I.Rastelli, EugenioRibet, DavidRatajczak, MagdalenaRicca, EzioRather, IrfanRichards, GaryRathinam, Navanietha KrishnarajRichardson, ElisabethRatkowsky, DavidRichardson, SarahRauceo, Jason M.Richter, LukaszRaudabaugh, DanielRichter, PeterRautelin, HilpiRiddick, Eric WellingtonRavaioli, StefanoRiehle, MichaelRavin, Nikolai V.Rietsch, ArneRawel, HarshadraiRinninella, EmanueleRawling, MarkRival, AlainRay, Jessica LouiseRiveiro-Barciela, MarRazafindralambo, Hary L.Rivero-Juárez, AntonioRazzuoli, ElisabettaRivilla, RafaelRedding, Kevin E.Rizzo, CarmenReddy, PriyankaRoana, JaniraReddy, SakamuriRobas, MarinaReddy, V. PrakashRobazza, Weber Da SilvaRedekar, NeelamRobb, FrankReed, David W.Robinson, HarrietReed, Kevin F.M.Roche, BenjaminReeves, MatthewRoche, RonanReffuveille, FanyRödelsperger, ChristianRego, RyanRodrigues, JoanaReguera, BeatrizRodrigues, Maria ElisaRehman, Md TabishRodrigues, Pedro MiguelRehnlund, DavidRodriguez Jovita, MarReis, CristianoRodríguez, AliciaReis, MárioRodríguez, MiguelRementeria, AitorRodriguez-Calvo, TeresaRemnant, EmilyRodríguez-Llorente, Ignacio DavidRendueles, ManuelRodríguez-López, PedroRepetto, OmbrettaRodríguez-Nogales, AlbaRequena, Jose MRodriguez-Rubio, LorenaRequicha, JoaoRodziewicz-Motowidło, SylwiaResa-Infante, PatriciaRoig-Sagués, ArturResch, BernhardRojas, AntoniaRestivo, Francesco MariaRojas, LauraReuter, KlausRojo, Maria AngelesReuter, TimRolbiecki, LeszekRevilla, EugenioRolinec, MichalRey, LuisRomano Spica, VincenzoReyes-Batlle, MaríaRomanò, Carlo L.Reyes-Ramírez, FranciscaRomano, JuliaReynard, OlivierRomano, PatriziaRho, HyungminRomanyà, JoanRial, RaquelRomberger, StevenRibaldone, DavideRomby, PascaleRomerio, FabioRymowicz, WaldemarRomero, AlejandroRyo, AkihideRomero-Romero, SoniaRyu, SangryeolRömling, UteRywińska, AnitaRomoli, OttaviaRząsa, AnnaRoncada, PaolaSabbatini, SamueleRoncevic, TomislavSacchetti, LuciaRong, Chan KuanSacchi, Gian AttilioRonsard, LaranceSachio, TsuchidaRosa, Bruce A.Sachithanandham, JaiprasathRosado, TâniaSadaoka, TomohikoRoscini, LucaSadykov, Marat R.Roseanu Constantinescu, AncaSagi, OrliRosen, BarrySaha, RanajayRosenke, KyleSaha, SunandanRosenthal, KenSaha, Sushanta KumarRoss, ElizabethSahay, BikashRoss, IanSahoo, Malaya KumarRossi, ChiaraSaint-Criq, VincianeRossi, FrancaSaito, SatoshiRossi, MaddalenaSaitoh, KenjiRossi, OmarSaitoh, TakejiRothman, Jason A.Sakai, FumikazuRotolo, CaterinaSakamoto, KensakuRotondo, John CahrlesSakanaka, MikiyasuRoubalová, RadkaSakudo, AkikazuRoura, XavierSala, ClaudiaRoy, DenisSalaheen, SerajusRoy, Denis JSaláková, MartinaRoy, JulienSalamon, SylwiaRóżalska, BarbaraŠalamún, PeterRóżańska, AnnaSalata, CristianoRuaro, BarbaraSaleem, MuhammadRudrappa, MohanSalehzadeh-Yazdi, AliRuiu, LucaSalek, KarinaRuiz Jimenez, AntonioSalem, MohamedRuiz Larrea, FernandaSalvador, AndreiaRuiz-Rico, MaríaSalvatore, PaolaRumbos, ChristosSalvatore, SilviaRumbou, ArtemisSamarasinghe, Amali E.Rupnik, MajaSampaio, AnaRusso, AlessandroSampaio, José PauloRusso, PasqualeSampedro, NagoreRusso, RosarioSamuelson, JohnRuta, LaviniaSamylina, Olga S.Ruzek, DanielSánchez Crespo, MarianoRyan, FrankSánchez Thevenet, PaulaRybak, AleksandraSánchez Torres, PalomaRybczyńska-Tkaczyk, KamilaSanchez Vargas, Luis AlbertoRychlik, IvanSánchez-Hidalgo, MarinaRyckman, BrentŠanderová, HanaSandle, TimSbrana, CristianaSandu, IonScanlon, Karen M.Sanjeewa, Kalu Kapuge AsankaScarano, AntonioSankaran, Ganapathy SubramanianScarborough, MatthewSanmartín, PatriciaScarlato, VincenzoSannino, CiroScarpa, FabioSanso, Andrea MarielScarpellini, EmidioSansone, AnnaScaturro, MariaSantacroce, LuigiSchaaper, RoelSantana, MargaridaSchabussova, IrmaSantander, JavierSchaumburg, FriederSantaniello, AntonioScheffel, AndréSantarem, NunoSchejter, EduardoSantero, EduardoSchempp, Christoph M.Santiago, Araceli E.Schena, LeonardoSantiago-Rodriguez, TashaSchifferli, Dieter MSantiago-Vázque, LorySchildgen, OlivierSantic, MarinaSchimel, JoshuaSantini, AntonelloSchindler, DanielSantos, CledirSchipper, KerstinSantos, FernandoSchlömann, MichaelSantos, JeffersonSchmacht, MaximilianSantos, Jesús A.Schmidt, AmySantos, Pedro M.Schmidt, HerbertSantos, RicardoSchmidt, MarcinSantos, SusanaSchmidt, MartinSá-Pessoa, JoanaSchmidt, RebeccaSapi, EvaSchmitt, EmmanuelleSapountzis, PanagiotisSchneegurt, MarkSaracino, IlariaSchneider, BarbaraSaraiva, Lígia M.Schoenian, GabrieleSaralahti, AnniSchoepp-Cothenet, BarbaraSaravanakumar, KandasamyScholz, MiklasSarbini, ShahrulSchotthoefer, Anna M.Šarkanj, BojanSchouten, AlexanderSarkar, AbhijitSchrallhammer, MartinaSarmiento, LuisSchroeder, Bjoern O.Sarshar, MeysamSchubert, NadineSase, AjinkyaSchughart, KlausSass, HenrikSchultz, David JSatoh, KatsuyaSchulz, StefanieSattley, W. MatthewSchuren, FrankSauka, DiegoSchütz, Luís FernandoSaul, DominikSchütz, LukasSauvage, ThomasSchwan, WilliamSavary, RomainSchwarz, LukasSavino, FrancescoSchweigkofler, WolfgangSawa, TeijiSchweizer, MagaliSawbridge, Timothy I.Schweizer, MichaelSawers, R. GarySchwermer, HeinzpeterSaxena, VikasSclavi, BiancaScobie, LindaShamputa, Isdore CholaScoffone, Viola CamillaShang, QingsenScudiero, OlgaShanmugam, GnanendraScully, Sean MichaelShanmugasundaram, RevathiSeaton, FionaShapaval, VolhaSecchi, EleonoraShapiro, Jason W.Sechi, Leonardo A.Sharifan, HamidrezaSecott, TimSharma, AtashiSedláček, DaliborSharma, CheshtaSedlacek, PetrSharma, DeepikaSee, Pao TheenSharma, ViragSeeman, Mary V.Shcherbakova, Larisa A.Seitz, JochenShcherbakova, ViktoriaSękara, AgnieszkaShchyogolev, SergeiSelan, LauraSheen, Shyn-ShinSeligmann, HerveSheikh, AlaullahSellin, MikaelShelake, Rahul MahadevSemenov, Mikhail V.Shelenkov, AndreySemenov, VyacheslavShelton, Chasity M.Sen Santara, SumitShemesh, MosheSen, KeyaShen, YangSengupta, AditiShen, ZhenyuŞenilă, MarinShenoy, Anukul T.Senwo, ZacharyShete, VarshaSeo, Ho SeongShetty, Kateel G.Seppey, ChristopheShi, RuiSequeda-Castañeda, Luis G.Shields-Menard, SaraSereikaite, JolantaShilev, StefanSereno, DenisShin, DonghyukSergeeva, Oksana A.Shin, Ho-joonSergiev, PetrShin, Jae HyunSergkelidis, DaniilShin, Jae-HoSério, SusanaShin, MinkyoungSerra, Claudia R.Shinde, TanviSerra, DiegoShinozaki, YukikoSerrano, Dolores R.Shintani, MasakiServín-Garcidueñas, Luis EduardoSHIRO, SokichiSerwer, PhilipShkryl, Yuri NSessa, RosaShmakova, LyubovSeuront, LaurentShoji, ToshihikoSevilimedu Veeravalli, SathyanarayananShort, Steven M.Sevvana, MadhumatiShree, SonalSeydel, KarlShrestha, BhushanSeydlová, GabrielaShrestha, NamitaShackelford, JuliaShrivastava, AbhishekShah, AjitShurpik, DmitriyShahi, Shailesh K.Shymanovich, TatsianaShaikh, SibhghatullaSichero, LauraShakerley, NicoleSidorenko, SergeyShakya, Viplendra P.S.Sieira, RodrigoShakya, Viplendra Pratap SinghSiemens, NikolaiSigillo, LoredanaSluder, Ann E.Sikora, Richard A.Šmajs, DavidSiltz, Lauren A.Smani, YounesSilva, MaraSmanski, MichaelSilva, Marcelo SousaSmedile, FrancescoSilvan, Jose ManuelSmet, CindySilvestre, RicardoSmith, DavidSilvestris, NicolaSmith, HillarySimo, LadislavSmith, KimothySimões, Marta FilipaSmith, MicholasSimon, EdurneSmith, NicholasSimpso J.Smits, SanderSinden, RichardSmokvina, TamaraSinderen, Douwe vanSmolander, Olli-PekkaSingan, Vasanth R.Smułek, WojciechSinger, Bernhard B.Smyth, BeatriceSinger, DavidSmyth, LeonSinger, Mitchell H.Snyder, LoriSingh, IshwarSoayfane, ZeinaSingh, Puspendra P.Sobral, OlimpiaSingh, RajbirSöderhäll, KennethSingh, SukhvinderSogorb Sánchez, Miguel ÁngelSinha, DebottamSoina, Vera S.Sinha, RohitaSolanki, Manoj KumarSinigaglia, Milena Grazia RitaSolano, FranciscoSiniscalco, DarioSolovyev, NikolaySioofy-Khojine, Amir-BabakSolyanikova, InnaSirén, KimmoSomlyai, GáborSiupka, PiotrSomma, StefaniaSivalingam, PeriyasamySonawane, JayeshSjöling, ÅsaSong, HaoSjöqvist, ConnySoo Ko, KwanSjostedt, AndersSorensen, JohnSkaradzińska, AnetaSoria Garcia, Juan MiguelSkellam, ElizabethSorrentino, E.Skladanka, JiriSosio, MargheritaSklodowska, AleksandraSoto, ManuelSkoneczny, SzymonSou, KeitaroSkorko-Glonek, JoannaSousa, Maria C.Skovgaard, OleSousa, Sílvia A.Skowron, KrzysztofSozhamannan, ShanmugaSkrede, SteinarSpadaro, SavinoSkrlep, MartinSpano, GiuseppeSkruzny, MichalSparrer, Konstantin M.J.Skrypnik, KatarzynaSpengler, JessicaSlaby, BeateSperfeld, MartinSlamova, KristynaSpiers, AndrewSlate, AnthonySpina, FedericaSleutels, TomSpinler, Jennifer K.Śliżewska, KatarzynaSplichal, IgorSlootweg, Erik JSplichalova, AllaSpormann, Alfred M.Striedner, GeraldSreenivasan, ChithraStromberg, ZacharySreevatsan, SrinandStrugnell, Richard A.Sridhar, SiddharthStrungaru, Stefan-AdrianSrikumar, ShabarinathStruzik, JustynaSriwastva, Mukesh KumarStulberg, MichaelStadlbauer-Köllner, VanessaStupica, DašaStaedler, DavideSturtevant, JoyStafford, James L.Styczyński, JanStahl, FelixSuárez, BelénStakos, DimitriosSuárez-Estrella, F.Stamatas, GeorgiosSubramanyam, BhadrirajuStana, AncaSucci, MariantoniettaStanciu, OanaSuda, ShoichiroStanford, KimSudar, MartinaStanford, StephanieSudhagar, ArunStanislauskienė, RūtaSue, Shih-CheStarčević, AntonioSugimoto, MitsushigeStark, JonnySui, YongjunStaroverov, SergeySul, WoojunStasiak-Różańska, LidiaSulik, ArturStasiewicz, Matthew J.Sullivan, ChrisStavrou, SpyridonSultana Rekha, RokeyaStearns, Jennifer CSulżyc-Bielicka, ViolettaStefanakis, AlexandrosSummerfield, TinaStefanello, SílvioSummers, Katie LynnStefani, FranckSun, FengjieStefanini, IreneSun, JiadongSteger, GerhardSun, KunfengSteinmann, EikeSUN, MingKuanStendahl, OlleSun, XiaolunStepanova, EkaterinaSun, YvonneStephens, EdwardSundberg, Lotta-RiinaStępień, ŁukaszSundheim, LeifSterkel, AlanaSung, KidonSterken, MarkSungthong, RungrochStervbo, UlrikSunna, AnwarStevens, David ColeSunter, Jack D.Stewart, George C.Superti, FabianaStewart, Julia M.Suter, AndyStines-Chaumeil, ClaireSuzutani, TatsuoStingl, Ulrich (Uli)Svenningsen, Sine LoStirke, ArunasSvircev, Antonet M.Stoffel, Martin A.Svoboda, LaurieStoian, VladSwidergall, MarcStoklosa, RyanŚwiętnicki, WiesławSt-Pierre, BenoitSydow, MateuszStrand, Micheline K.Sylte, Matthew J.Strati, KaterinaSynowiec, AgnieszkaStrbak, OliverSyromyatnikov, Mikhail Y.Street, Teresa L.Syron, EoinSytykiewicz, HubertTao, YunwenSzacawa, EwelinaTaoka, YousukeSzafranski, SzymonTap, JulienSzandruk, MartaTarlinton, RachaelSzara, EwaTarrah, ArminSzatmari, AgnesTartera, CarmenSzili-Kovács, TiborTascini, CarloSzkaradkiewicz, AndrzejTashima, ToshihikoSzkuta, BiancaTatu, Alin LaurenţiuSzostakowska, BeataTauber, JamesSzóstek-Mioduchowska, AnnaTavares, JoanaSzulc, PiotrTaverniti, ValentinaSzumacher-Strabel, MalgorzataTaylor, MartinSzweda, PiotrTaylor, MatthewSzymanowska-Powałowska, DariaTaylor, NicholasSzymańska-Czerwińska, MonikaTe Velthuis, AartjanSzymanski, ChristineTechjian, GildatTaank, VikasTeixeira-Santos, RitaTabacchioni, SilviaTelle-Hansen, Vibeke H.Tabanelli, GiuliaTellez-Lance, AngelaTabara, MidoriTelling, JonTachibana, ShinjiroTemesvari, LeslyTadych, MariuszTender, Caroline DeTafforeau, LionelTenenbaum, TobiasTagad, HarichandraTeng, ShaoleiTagele, Setu BazieTenhagen, Bernd-AloisTagliavia, MarcelloTeramoto, TadahisaTaguchi, KenseiTeras, RihoTaheri, ShimaTerhonen, EevaTai, Dar-InTerzi, ValeriaTai, WanboTesei, DonatellaTakaki, KoichiTesh, VernonTakata, TomoakiTetz, GeorgeTakeda, MakotoTetz, VictorTakeda, MinoruThammina, Chandra SekharTakemoto, JonThangamani, ShankarTakemoto, KazuhiroThangavel, TamilTakeyama, HarukoTharmalingam, NagendranTakors, RalfThe, ErlindaTall, Ben D.Thekkiniath, JoseTalon, RegineThevissen, KarinTamori, AkihiroThielen, BethTampakaki, AnastasiaThierry, AlainTamura, TomohikoThies, StephanTan, Bee KThomas, Donald B.Tang, YaoThompson, AndrewTani, YukinoriThompson, JeremyTanner, JeromeThompson, NickTannières, MélanieThompson, Stuart A.Tao, PanThorpe, ClareTao, PengTian, RenmaoTichaczek-Goska, DorotaTraina, GiovannaTidona, FlavioTran, Seav-LyTilocca, BrunoTråvén, MadeleineTimling, InaTrcek, JanjaTimofeev, Vladimir I.Trček, JanjaTirelli, AntonioTremblay, Émilie D.Tirloni, EricaTresse, OdileTiwari, PoojaTretina, KyleTkacz, AndrzejTreusch, Alexander H.Tkadlec, JanTrilling, MirkoTkavc, RokTrinchera, MarcoTlaskalová-Hogenová, HelenaTristram, StephenTodd, LaurenTrögl, JosefTodorov, Svetoslav DimitrovTropea, AlessiaTodt, DanielTrouvelot, SophieTofalo, RosannaTroyer, RyanToiu, AncaTruu, JaakTokach, MikeTsai, An-YiTomasevic, IgorTsai, Kun-HsienTomaszewska, EwaTsakraklides, VasilikiTomczyk, ŁukaszTsaltas, DimitrisTomita, HaruyoshiTschirhart, TanyaTompkins, Thomas A.Tse, HubertTonelli, MicheleTseng, Kuang-WenToniolo, AntonioTsiknia, MyrtoToniolo, Antonio Q.Tsioutis, ConstantinosToovey, StephenTsirigotis, PanagiotisTorija, Maria JesúsTsugawa, HitoshiTormo, JoseTsuji, MasaharuTorović, LjiljaTsumuraya, TakeshiTorquati, LucianaTuaillon, EdouardTorregrosa-Crespo, JavierTufariello, MiriamTorren, Laura VilanovaTullman-Ercek, DanielleTorres Barcelo, ClaraTumanov, Alexei V.Torres Fuentes, CristinaTuovinen, OlliTorres Sangiao, EvaTurchetti, BenedettaTorres, CarmenTurgay, KürsadTorres-Perez, JuanTurnau, KatarzynaTorres-Sangiao, EvaTurroni, SilviaTorri, EmanueleTurton, JaneTortosa, GermánTurton, Jane FTorvinen, EilaTurzo, KingaToshchakov, StepanTyurin, Anton P.Toshimitsu, TakayukiTzanakakis, VasileiosTóth, Gábor K.Tzeng, Tzuen-RongTouraki, MariaTzima, Aliki K.Town, JenniferUcciferri, ClaudioToyoma, TadashiUchil, Pradeep D.Toyoshima, MasakazuUchiyama, SatoshiTrabelsi, KhaledUddin, Md NazimTragust, SimonUebanso, TakashiUeda, AkihiroVarela, CristianUeno, AkioVarga, LászlóUgalde, Juan E.Varma, Rajender S.Ulrich, KristinaVaroni, Elena MariaUlusoy, BeyzaVarzakas, TheodorosUnno, TatsuyaVashist, SandeepUpdegrove, Taylor B.Vashist, Sandeep KumarUrban, ConstantinVasina, Daria V.Urbán, EditVater, AshleyUrban, PhilippeVavitsas, KonstantinosUrbańczyk-Lipkowska, ZofiaVazquez Belda, BeatrizUrbani, AndreaVázquez-Campos, XabierUrbaniak, MonikaVeiga, PatrickUrbieta, María SofíaVelay, AurélieUsai, DonatellaVelena, AstridaUsher, JaneVelikova, TsvetelinaUyeno, YutakaVellios, EvangelosV’kovski, PhilipVemula, HarikaVachiraroj, NuttaponVemuri, RavichandraVágvölgyi, CsabaVenieraki, AnastasiaVahjen, WilfriedVenkannagari, HarikanthVaishampayan, ParagVenter, HenriettaValdés López, OswaldoVenturi, ManuelValentine, Christina J.Verdenelli, CristinaValenzuela-fernández, AgustínVeres, SzilviaValiakos, GeorgeVergunst, Annette C.Valitutti, FrancescoVerma, RanjitVallesi, AdrianaVerma, SmritiValm, Alex M.Verran, JoannaValverde, ClaudioVerruck, SilvaniVamanu, EmanuelVestby, Lene KarineVan Belleghem, Jonas D.Vetvik, KatjaVan Calsteren, Marie-RoseViana, FlaviaVan Den Hoogen, BernadetteVideira E Castro, IsabelVan Den Hurk, AndrewVidican, RoxanaVan Der Kuyl, AntoinetteVidovic, SinisaVan Der Vaart, MichielVieira, SelmaVan Der Ven, André J.Am M.Vieira-Pinto, MadalenaVan Der Werf, T.S. (Tjip)Viejo-Borbolla, AbelVan Der Wielen, Paul W. J. J.Vieyra, Jorge ParedesVan Etten, JamesVigentini, IleanaVan Kessel, AndrewVigilato, Marco Antonio NatalVan Kranenburg, RichardVijgenboom, ErikVan Straalen, Nico M.Vikram, AmitVan Wyk, NielVilain, SébastienVan, Thi Thu HaoVílchez-Vargas, RamiroVanaverbeke, JanVilela, AliceVanEpps, J. ScottViles, HeatherVanlandingham, DanaVilla, FedericaVanneste, Joel L.Villa, Tomas G.Varaldo, Pietro E.Villalba Martínez, RubinVillari, SaraWang, PengchengViñas, MiguelWang, Robert Y.-L.Vinayak, SumitiWang, ShaobinVincent, AntonyWang, ShaohuaVinuesa, TeresaWang, Sheng-FanVipperla, KishoreWang, ShihuaVirok, Dezső P.Wang, SishuoVirolle, Marie-JoelleWang, XiuqinVisca, PaoloWang, ZenengVishnivetskaya, Tatiana A.Wang, ZhejunVismarra, AliceWang, ZhuoVitale, MariaWard, Honorine D.Vitalii, TimofeevWard, SelinaVitas, Ana IsabelWareth, GamalVitetta, LuisWasilkowski, DanielViviani, SimonettaWassenaar, Trudy M.Voběrková, StanislavaWatabe, ShugoVoermans, Jolanda J.C.Watanabe, TokikoVogel, Hans J.Watier-Grillot, StéphanieVogel, KevinWauters, LucasVogt, MeganWawiórka, LeszekVoidarou, ChrysaWeadge, JoelVon Bergen, MartinWeber, BarbaraVon Herrat, Matthias G.Weber, J. MarkVon Wettstein-Knowles, PennyWedwitschka, HaraldVoronina, O. L.Weger, StefanVriesekoop, FrankWegrzyn, AlicjaVučković, DarinkaWegrzyn, GrzegorzVukušić Pavičić, TomislavaWęgrzyn, GrzegorzVuotto, ClaudiaWeiland-Bräuer, NancyVurukonda, Sai Shiva Krishna PrasadWeimer, PaulVuyst, Luc DeWeinhold, ArneVylkova, SlavenaWeiss, SiegfriedWacławek, StanisławWelliver, RobertWade, MatthewWemheuer, BerndWadsworth, CristaWemheuer, FranziskaWagemans, JeroenWendisch, Volker F.Wajs-Bonikowska, AnnaWendling, DanielWaldminghaus, TorstenWerner, JohannesWałecka-Zacharska, EwaWertz, Philip W.Waleron, MałgorzataWessely-Szponder, JoannaWall, DanielWestreicher-Kristen, EdwinWalter, MonikaWestwater, CarolineWalther-Antonio, MarinaWheeler, Terry A.Wang, Fun-InWhite, JohnWang, GuorongWhite, Richard AllenWang, HaitaoWhite, SimonWang, Jian-WenWhitehurst, Christpher B.Wang, Jiun-LinWhitten, SteveWang, JunwenWidemann, EmilieWang, LeyiWidmer, Giovanni F.Wieczfińska, JoannaXu, LingWielbo, JerzyXu, ZhenboWierckx, NickYadav, VinitaWietz, MatthiasYaghoubi Khanghahi, MohammadWiewióra, BarbaraYagüe, PaulaWilharm, GottfriedYamada, HiroyukiWille, MichelleYamamoto, MasakiWilletts, AndrewYamamoto, NaomichiWilliams, E. MarkYamamoto, TatsuyukiWilliams, KevinYamanaka, TakashiWilliams, LisaYamasaki, ShoWilson, WilliamYamashiro, YuichiroWinans, StephenYan, QiangWinter, JaclynYan, ZiyingWise, Michael J.Yanagisawa, NaokoWiśniewska, MałgorzataYang, Chu-WenWisniewski, MichaelYang, ShangxinWisselink, HenkYang, YangWiszniewska-Łaszczych, AgnieszkaYang, ZhaohaiWitola, William HaroldYang, ZimingWitrick, KatherineYao, ChaoqunWitte, AngelaYao, YiqingWlazło, ŁukaszYaoita, YasunoriWłoga, DorotaYaraki, Mohammad TavakkoliWnuk, MaciejYarbrough, WendellWojdyło, AnetaYaron, SimaWojtkowska, MałgorzataYassin, MohamedWojtyczka, RobertYavitt, JosephWolf, MatthiasYedidia, IrisWolfrum, Edward J.Yee, Wee L.Wolińska, AgnieszkaYeh, Kuang-ShengWolz, ChristianeYekta, Sepehr ShakeriWooten, MarkYeoman, CarlWorwa, GabriellaYeruva, LaxmiWöstemeyer, JohannesYilmaz, BahtiyarWu, ChaoYin, ChangchuanWu, GuanghuiYin, ChaoranWurster, SebastianYin, JieWysok, BeataYin, MengWyszkowska, JadwigaYlostalo, Joni H.Xavier, Joana C.Yokota, AkiraXia, FangYokoyama, HiroshiXiao, Jin-zhongYokoyama, KazumasaXie, JieYonekura, ShinichiXie, WankunYoo, Han SangXin, YinYoo, KeunjeXiong, WuYoshinari, TomoyaXu, ChuanYoshioka, Ken-ichiXu, JianYoshizaki, TomokazuXu, JinYou, HyunjuXu, JintaoYu, GangYu, Hong-RenZeng, MelodyYu, JianmeiZengler, KarstenYu, JingyouZervas, AthanasiosYu, MandaZhang, ChangyiYu, Ollie YiruZhang, DuoYu, ZhongtangZhang, FanYuan, ZhihongZhang, HongjieYukl, Erik T.Zhang, JileiYuko, NishimotoZhang, LixinYumoto, IsaoZhang, MingYun, Cheol-WonZhang, PengYurchenko, EkaterinaZhang, QiongYurimoto, HiroyaZhang, RuiyongYushenova, IrinaZhang, ShuYuste, JoseZhang, TianyuYusuf, ErlanggaZhang, XiaotaoYuval, BoazZhang, YijunZacariah, HildenbrandZhang, YinfengZachariah, RonyZhang, YongliangZacharioudakis, Ioannis M.Zhao, XihongZago, VanessaZhou, ManZaharescu, Dragos G.Zhou, MiZaini, PauloZhu, ChangfuZaiter, AliZhu, JingenZając, MagdalenaZhu, KuiZając, ZbigniewZhu, LuchangZambelli, BarbaraZhu, ShiweiZambrini, Angelo Stavro VittorioZhu, XiaochengZamfir, MedanaZhukov, Vladimir A.Zanella, AugustoZhukova, NataliaZanella, IsabellaZiarno, MałgorzataZanetti, StefaniaZielińska, DorotaZara, GiacomoZieniuk, BartłomiejZara, SeverinoZifcakova, LuciaZaragoza, WilliamZiganshin, AyratZarfel, Gernot E.Ziganshin, Ayrat M.Zarrilli, RaffaeleZimba, PaulZavilgelsky, Gennadii B.Zimcik, PetrZawilak-Pawlik, Anna MagdalenaZimmerman, TahlZawrotniak, MarcinZimmermann, Sabine DagmarZdarta, AgataZipprich, HolgerZdolec, NevijoZitong, LiZdybicka-Barabas, AgnieszkaZlatan, MujagicZechiedrich, LynnZmora, PawelZeigler, DanielŻołek, TeresaZeineldin, MohamedZolla, LelloZeleny, ReinhardZolnik, Christine P.Zell, RolandZomer, Aldert L.Zella, DavideZotta, TeresaZeman, PetrZou, ChunbinZendo, TakeshiZou, WeiZuber, PeterŽunar, BojanZucconi, LauraZuniga, CristalZuffo, MichelaZuñiga, SoniaZumstein, Michael ThomasZygadlo, Julio Alberto

